# Targeting β-catenin and PD-L1 simultaneously by a racemic supramolecular peptide for the potent immunotherapy of hepatocellular carcinoma

**DOI:** 10.7150/thno.83377

**Published:** 2023-06-04

**Authors:** Zhengjun Zhou, Xiao Li, Guang Yang, Jingmei Wang, Baohua Li, Yinong Huang, Jin Yan, Kaishan Tao

**Affiliations:** 1 Department of Hepatobiliary and Pancreaticosplenic Surgery, Xijing Hospital, Air Force Medical University (The Fourth Military Medical University), Xi'an 710032, China.; 2 National & Local Joint Engineering Research Center of Biodiagnosis and Biotherapy, The Second Affiliated Hospital of Xi'an Jiaotong University, Xi'an, 710004, China.; 3 Department of Oncology, Suzhou BenQ Medical Center, The Affiliated BenQ Hospital of Nanjing Medical University, Suzhou, Jiangsu Province, China.; 4 Institute for Stem Cell & Regenerative Medicine, The Second Affiliated Hospital of Xi'an Jiaotong University, Xi'an 710004, China.; 5 Core Research Laboratory, The Second Affiliated Hospital of Xi'an Jiaotong University, Xi'an 710004, China.; 6 Shaanxi Institute of Pediatric Diseases, Xi'an Children's Hospital, Xi'an, 710003, China.

**Keywords:** supramolecular peptide, β-catenin, PD-L1, tumor immunotherapy, hepatocellular carcinoma

## Abstract

**Objective:** The low clinical utility of immune checkpoint inhibitors (ICIs) against PD-1 or PD-L1 has recently been associated with the activation of the Wnt/β-catenin signaling pathway in hepatocellular carcinoma (HCC), which promotes tumor immune escape and resistance to anti-PD-1/PD-L1 therapy. Hence, we aimed to fabricate a supramolecular peptide which could target the Wnt/β-catenin signaling pathway coupled with ICIs blockage therapy for optimizing HCC immunotherapy.

**Methods:** A racemic spherical supramolecular peptide termed sBBI&PDP nanoparticle was constructed by hierarchical self-assembly, comprising an L-enantiomeric peptide as an inhibitor of BCL9 and β-catenin (sBBI) and a D-enantiomeric peptide as an inhibitor of PD-1/PD-L1 (PDP).

**Results:** sBBI&PDP nanoparticle potently suppressed the hyperactivated Wnt/β-catenin signaling pathway *in vitro* and *in vivo*, while blocking endogenous PD-L1 effectively. Furthermore, sBBI&PDP increased the infiltration and action of CD8^+^ T cells at tumor sites. Notably, compared with the original sBBI and commercial Anti-PD-L1 inhibitors, the designed sBBI&PDP showed stronger antitumor efficacy in an orthotopic homograft mice model of HCC and a PDX HCC model in Hu-PBMC-NSG mice. Moreover, sBBI&PDP possessed a favorable biosafety profile.

**Conclusion:** The successful implementation of this strategy could revitalize ICIs blockage therapy and promote the discovery of artificial peptides for HCC immunotherapy.

## Introduction

Hepatocellular carcinoma (HCC) poses a serious threat to human health, and according to the prediction of the World Health Organization, one million people will die from liver cancer by 2030 1. For patients with advanced liver cancer, the prospect for a cure and long-term survival of HCC remains dim 2. In spite of the fact that immune checkpoint inhibitors (ICIs) towards PD-1 or PD-L1 that have emerged in recent years have shown partial efficacy in HCC patients, the overall clinical effectiveness rate is low, accounting for only around 20% of patients 3. The low clinical benefit has recently been linked to the activation of Wnt/β-catenin signaling pathway in HCC 4, which promotes tumor immune escape and resistance to anti-PD-1/PD-L1 therapy 5-8. Specifically, tumors disrupt the immune response surveillance and cytotoxicity by using two major mechanisms mediated by the Wnt/β-catenin signaling pathway: 1) they promote the differentiation and activity of Treg cells, and 2) they minimize the infiltration of CD8^+^ T cells into the tumor microenvironment 9, 10. Moreover, the hyperactivated Wnt/β-catenin signaling pathway in HCC has been associated with an immunosuppressive phenotype of the tumor microenvironment characterized by impaired tumor infiltration of immune cells 11, 12, which presumably contributed to the low clinical response PD-1/PD-L1 immune checkpoint inhibitors 5. Thus, targeting the Wnt/β-catenin signaling pathway coupled with ICIs blockage therapy is a very promising strategy for optimizing tumor immunotherapy.

Nevertheless, the Wnt/β-catenin pathway is also required for stem cell proliferation and differentiation, which makes clinically beneficial therapeutic interventions a challenge 13. Fortunately, modulating the interaction between β-catenin and B-cell lymphoma 9 (BCL9) has recently made breakthroughs 14. BCL9, a key transcriptional co-activator of the Wnt pathway and highly expressed in tumors, activates the Wnt/β-catenin signaling pathway by interacting with β-catenin, promoting its nuclear translocation, and promoting tumor progression 15. Some studies have shown that BCL9 antagonists can inhibit tumor growth, promote CD8^+^ T cell infiltration of tumors, and enhance anti-PD-1 immune responses 16. Another group of researchers developed a peptide called hsBCL9_CT_-24 to reduce regulatory Treg cells and increase cytotoxic T cells by targeting and inhibiting the β-catenin/BCL9 interaction in a colorectal cancer (CRC) model 17. Thus, modulating the interaction between β-catenin and BCL9 by peptide is a promising strategy for the safe and efficient suppression of Wnt/β-catenin pathway.

At present, the development of peptide drugs targeting protein-protein interactions (PPIs) mainly faces problems such as the limited protease resistance, poor membrane permeability, and short blood circulation time. To address these issues, researchers locally altered the main-chain and side-chain peptidomimetics, disulfide bonds for conformational stabilization, encapsulation by nanomedicines to improve the spatial structure of the peptides 18-20, resulting in the improvement of peptide stability against enzymes and prolong their biological half-lives 21-23. Our previous work also demonstrated that assembling peptides with Au(I) to a tumor microenvironment (TME)-responsive nanoclusters can achieve the tumor-specific accumulation 24, 25, and this strategy was also appropriate for the delivery of PD1/PD-L1 checkpoint blockade (PPB) therapeutics 26-28.

**Figure 1:**
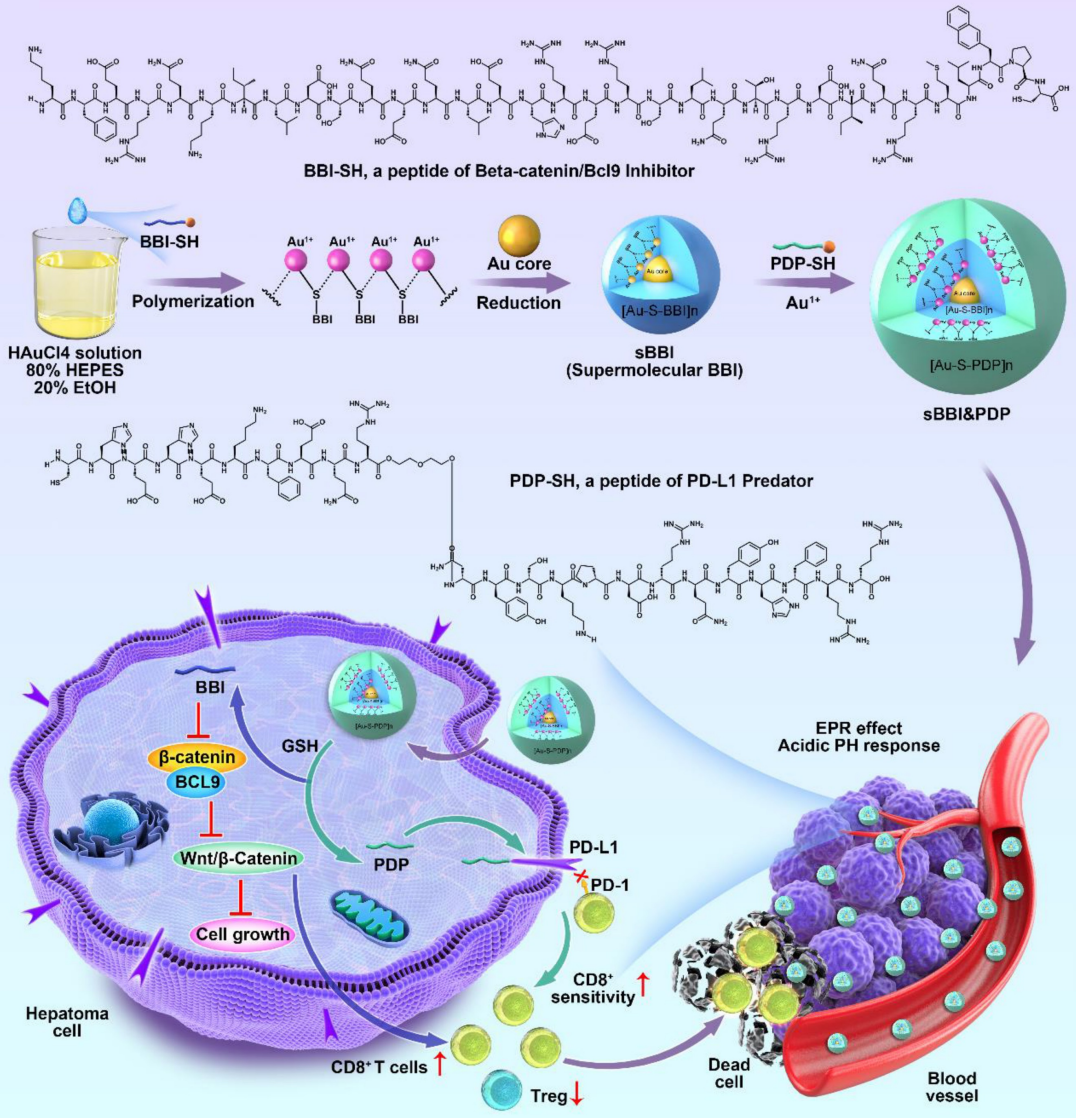
**Schematic depiction of sBBI&PDP synthesis and function for tumor-cytotoxic effect and inhibited growth of hepatocellular carcinoma**.

Herein, by hierarchical self-assembly, a racemic spherical supramolecular peptide termed sBBI&PDP was constructed, comprising an L-enantiomeric peptide as an inhibitor of BCL9 and catenin (sBBI) and a D-enantiomeric peptide as an inhibitor of PD-1/PD-L1 (PDP) (Figure 1). As expected, sBBI&PDP potently suppressed the hyperactivated Wnt/β-catenin pathway *in vitro* and *in vivo*, while blocking endogenous PD-L1 effectively. BBI prevented the interaction of β-catenin and BCL9 in tumor cells, inhibited the activation of the canonical Wnt/β-catenin signaling pathway, thereby inhibiting the growth of tumor cells and recruiting more CD8^+^ T cells to act on the tumor (Figure 1). On the other hand, PDP binds to the intracellular segment of PD-L1 molecules, blocks the interaction of PD-1/PD-L1, sensitizes drug-resistant CD8^+^ T cells, and enhances the effect of killing tumor cells (Figure 1). Notably, compared with the original sBBI, commercial Anti-PD-L1 or Wnt inhibitor, the designed sBBI&PDP showed stronger antitumor efficacy in an orthotopic homograft mice model of HCC and a PDX HCC model in hu-PBMC-NSG mice. Moreover, sBBI&PDP possessed a favorable biosafety profile. Collectively, the successful implementation of this strategy could revitalize ICB blockage therapy and promote the discovery of artificial peptides for HCC therapy.

## Results and Discussion

### The design and construction of sBBI&PDP

*s*BBI&PDP was constructed by a hierarchical self-assembly comprising an L-enantiomeric peptide as an inhibitor of BCL9 and catenin (BBI) and a D-enantiomeric peptide as an inhibitor of PD-1/PD-L1 (PDP). Notably, an additional right-handed Cys residue was introduced at the C-terminus of BBI and PDP, both of which were synthesized by FMOC solid-phase peptide chemistry 29-33 in about 70% yield with >95% purity by LC-MS (Figure S1A&S1B). HRMS data of BBI or PDP represented 4344.316 Da (Figure S1C) or 3433.659 Da (Figure S1D), respectively. Besides, And the abundant side chain functional groups in the 1H-NMR spectrum verified the successful preparation of BBI (Figure S1E) and PDP (Figure S1F). The hierarchical self-assembly of sBBI&PDP was driven by the aurophilic interactions 34-39. The aurophilicity was referred to the existence of specific intramolecular and intermolecular bonds between gold(I)(5d10) centers within closed surface shells, which was manifested in several fields of gold chemistry. In some cases, it was even related to the binding energy of strong hydrogen bonds, so it was very important to help determine molecular structure and dynamics. 40, 41. Besides, the cysteine of peptide was essential for peptide supramolecular assembly which was used to form stable Au-S bond 42. In a word, the aurophilic interaction was performed also among the Au(I)-peptide precursors [Au(I)-S-BBI]n or [Au(I)-S-PDP]n (Figure 1).

**Figure 2:**
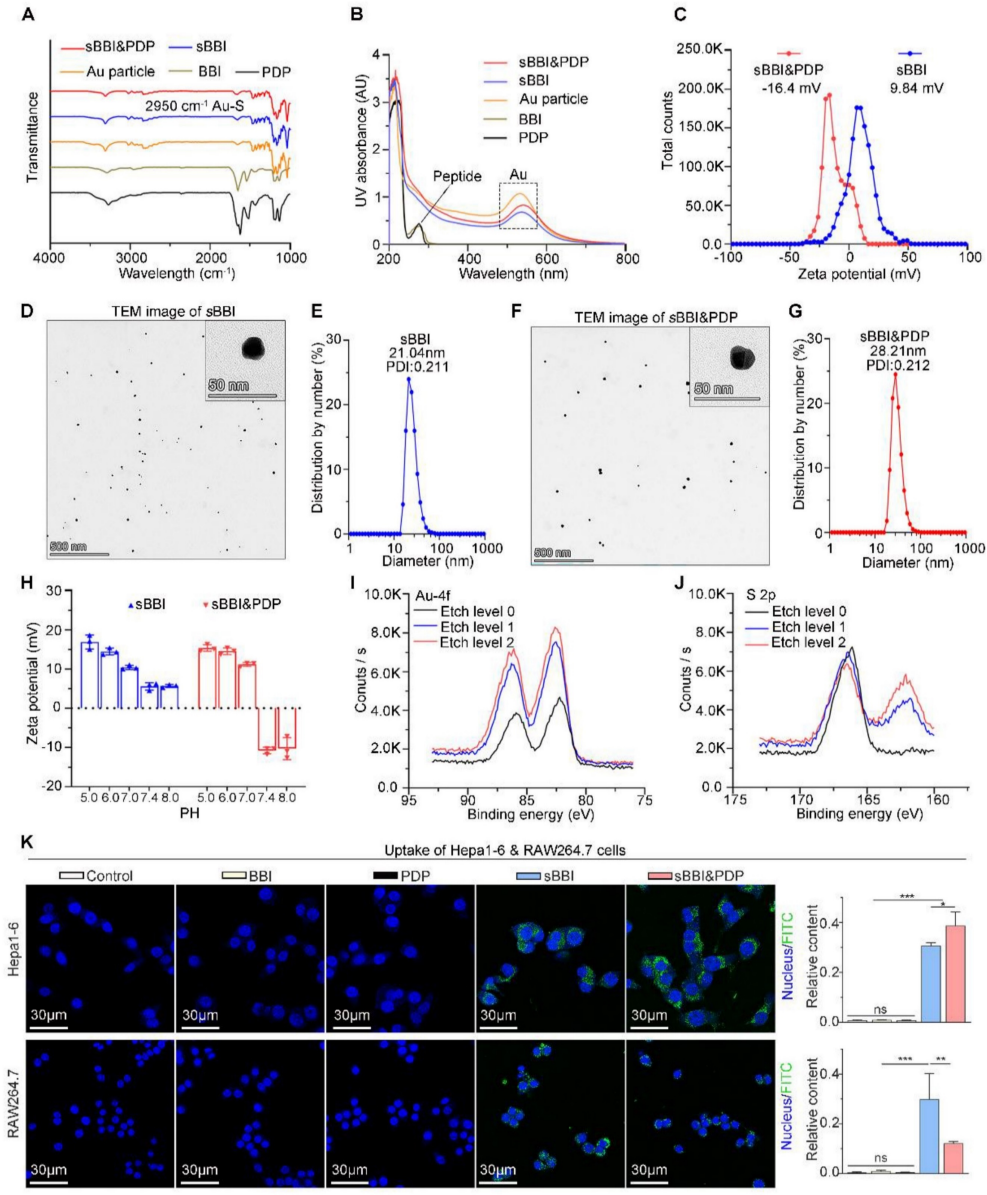
** Physicochemical properties of sBBI&PDP.** (A) FT-IR spectra of Au particle, free peptide (BBI or PDP) and Au-peptide polymeric structure (sBBI or sBBI&PDP). (B) UV-vis absorption spectra of sBBI&PDP, sBBI. Au particle, BBI or PDP. The red shift of the absorption peak near 530 nm revealed the increase of nano size of *s*BBI&PDP, compared with *s*BBI or Au particle. (C) Zeta potential of sBBI&PDP and *s*BBI measured in PBS at pH 7.4. (D, E) TEM image (D) and size distribution by number (E) of *s*BBI. (F, G) TEM image (F) and size distribution by number (G) of sBBI&PDP. (H) Charge reversal of *s*BBI&PDP compared with *s*BBI. (I, J) XPS spectra of Au 4f (I) and S 2p (J) at the surface and after etching of sBBI&PDP. (K) Uptake images and quantitative analysis of sBBI&PDP observed by confocal laser scanning microscope in the Hepa1-6 hepatoma cells or RAW264.7 macrophagocytes compared with sBBI, BBI, PDP or Control (*n* = 3, *t*-test, **p < 0.01; ***p < 0.001).

This self-assembly reaction was a "one-pot two-step reaction" under mild conditions. Step 1: mixed 1 mg BBI and 1 mg NH2-PEGn-SH (MW: 2000 Da) in the 5 mL of 20 mM HAuCl4 solution. Then 5 ml of 100 mM HEPES buffer (pH 7.4) was added to form sBBI nanoparticles. Next, after magnetic stirring for 5 minutes, 5 mL of 20 mM HAuCl4 solution containing 1 mg PDP was added in step 2, and the color of the solution turned purple. Mass concentration of BBI or PDP in the sBBI&PDP solution was 1 mg/15 mL, MW of BBI was 4344.316 Da and MW of PDP was 3443.659 Da by the high-resolution mass spectrometry (HRMS) (Figure S1C-D), converted to molar concentration by calculator, BBI: 15.346 μM, PDP: 19.359 μM, and then the molar ratio of BBI: PDP in sBBI&PDP nanoparticle was 1:1.262. Fourier transform infrared (FT-IR) spectrum (Figure 2A) confirmed the formation of the peptide-Au conjugation as evidenced by the properties of FT-IR of Au-SR absorbance. Ultraviolet visible (UV-vis) spectroscopy (Figure 2B) shown the formation of nanoparticles, as indicated by UV-vis absorption (540 nm), which was due to the plasmon resonance of the gold nanoparticles. As proof of the successful coating of PDP, the surface charge and particle size of sBBI and sBBI&PDP were measured; a negative shift in Zeta potential (Figure 2C) and an increase in particle size (Figure 2D-G) corroborated this result. And sBBI&PDP nanoparticles presented the spherical supramolecular gold(I)-thiol-peptide complexes (Figure S2A), and the energy-dispersive spectroscopy (EDS) analysis (Figure S2B) demonstrated an even distribution of Au, S, N, and O in sBBI&PDP suggesting the existence of Au-S-BBI or PDP. sBBI&PDP had a high loading efficiency of BBI or PDP peptides compared to gold nanoparticles (AuNPs) with two peptides at the same time by HPLC analysis (Figure S2C-D). Note that sBBI&PDP showed electronegativity at pH > 7.0 and electropositivity at pH < 7.0, whereas sBBI maintained its positive charge both in alkaline and acidic environments (Figure 2H). Moreover, X-ray photoelectron spectroscopy (XPS) of the intermediate between the peptide and Au(I) confirmed that the Au (4f) peak positions of the sBBI&PDP were in accordance with the results previously reported for Au(I) ions conjugated to alkanethiols in Cys residues (Figures 2I-J). Additionally, the colloid stability of sBBI&PDP was observed in the PBS solution containing 20% FBS by monitoring its hydrodynamic size, which nearly maintained its initial size with 168 hours (Figure S3).

Besides, we contrastively explored cellular uptakes of BBI, PDP, sBBI and sBBI&PDP into a macrophage cell line RAW264.7 and HCC cell line Hepa1-6. The fluorescence was derived from fluorescein isothiocyanate (FITC), FITC was linked to the amino-terminal of sBBI, and the sBBI with FITC was then combined with PDP to form FITC-sBBI&PDP. We demonstrated the binding of BBI peptide to FITC by HPLC and Mass (Figure S4). As shown in Figure 2K, neither BBI nor PDP peptide showed cellular uptakes, presumably because of the degradability or cytomembrane unpermeability. Moreover, sBBI&PDP showed the weakened cellular uptakes into RAW264.7 in comparison with sBBI (Figure 2K), which may be profited from its negative surface charge at pH 7.4. More importantly, PDP modification would increase the affinity between the nanoparticle and HCC cytomembrane, which enhanced the cellular uptakes of sBBI&PDP into Hepa1-6 cells comparing with sBBI (Figure 2K). By comparing the baseline level of PD-L1 in cells (Figure S5A), it was found that Hepa1-6 (21.1%) was higher than RAW246.7 (2.37%), and PDP, an antagonistic peptide of PD-L1, was more likely to bind to Heap1-6. Secondly, flow cytometry (Figure S5B) showed that after PD-L1 antibody was applied to the above two cells, the uptake of sBBI&PDP decreased to a certain extent, and the decrease was more obvious in Hepa1-6 cells than RAW264.7 cells. Additionally, polyethylene glycol (PEG)-modified nanoparticles would help to avoid macrophage clearance 43, and sequence of PDP was CHEHEKFEQR-(PEG3)-nyskptdrqyhfrr. The cell uptakes analysis demonstrated that specialized sBBI&PDP structures could target HCC cells and reduce their phagocytosis by macrophages. In summary, these results indicated that sBBI&PDP nanoparticles were successfully fabricated as the spherical supramolecular gold(I)-thiol-peptide complexes.

### The release of peptides and pharmacokinetics of sBBI&PDP

**Figure 3:**
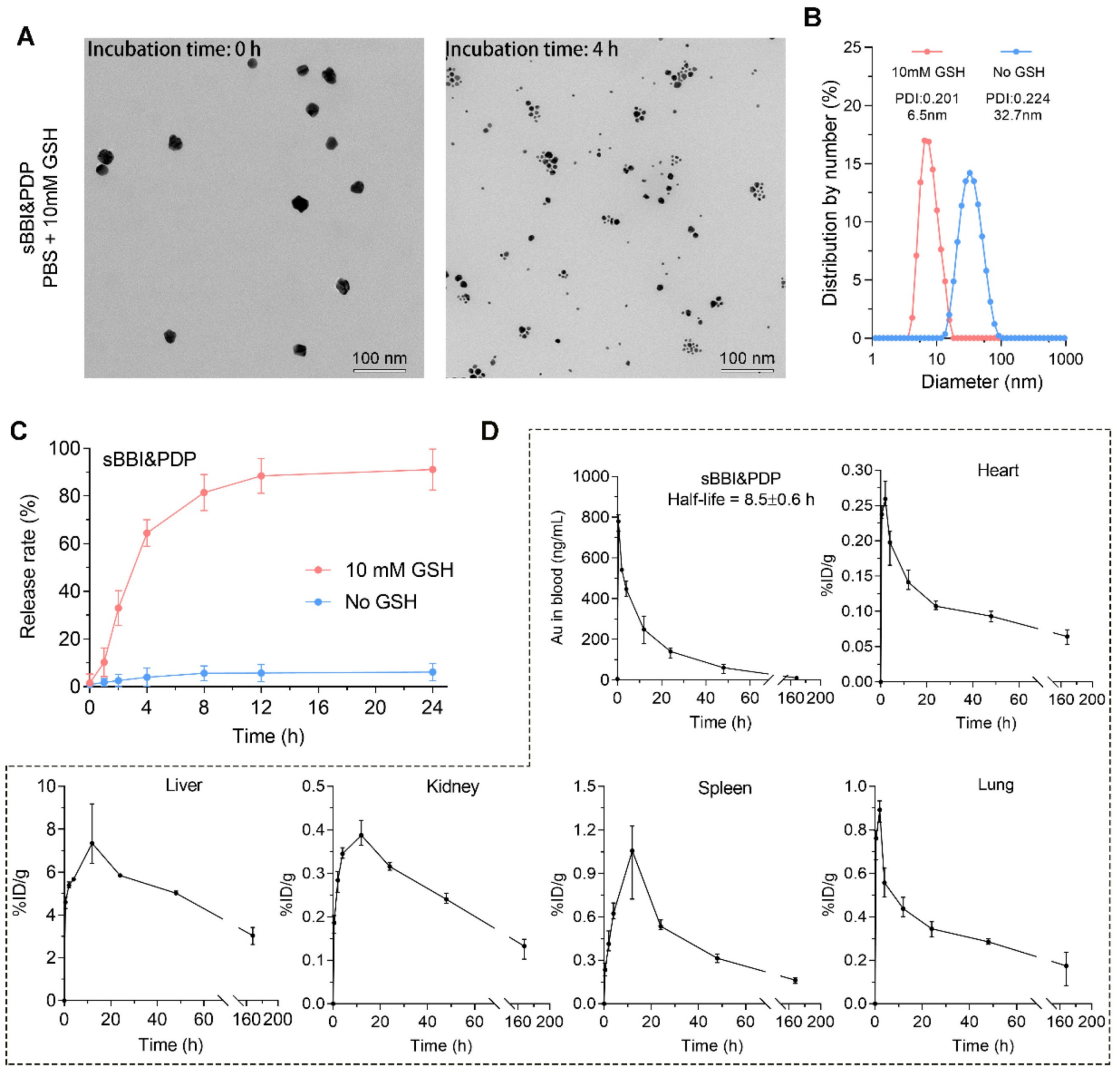
** The release of peptides and pharmacokinetics of sBBI&PDP**. (A, B) TEM image and hydrodynamic diameter of sBBI&PDP incubated in PBS solution with 10 mM GSH after 0 h or 4 h, respectively. (C) Peptides release from sBBI&PDP under 10 mM GSH or 10 μM GSH solution at pH 7.4. Peptide release was quantified by HPLC. (D) The pharmacokinetics of sBBI&PDP in C57BL/6J mice after tail vein injection. Serial sacrifices were carried out at 0 h, 0.5 h, 2 h, 4 h, 12 h, 24 h, 48 h and 168 h (1 week) after dosing. Several organs/tissues, including blood, heart, lung, kidney, spleen and liver were isolated to determine gold concentrations by ICP-MS. The data were shown as mean ± SD.

Glutathione (GSH) could trigger the release of thiols from the surface of AuNPs, and intracellular GSH concentration has been reported to be in the millimolar level, whereas extracellular GSH was found only in the micromolar range 44. More importantly, GSH concentration was usually elevated in tumor cells 45. Therefore, we assumed that the high GSH concentration within hepatoma cells could produce the disassembly of sBBI&PDP nanoparticles. As expected, the TEM size and diameter of sBBI&PDP nanoparticles became smaller when 10 mM GSH was added, suggested that sBBI&PDP were disassembled (Figure 3A-B). High-performance liquid chromatography (HPLC) assay also showed GSH-triggered peptides release from sBBI&PDP nanoparticles. There was a release order between two peptides, and the PDP in the outer layer of the sBBI&PDP nanoparticle was released first, followed by the BBI near the Au core. As shown in Figure 3C, sBBI&PDP was incubated in PBS buffer (No GSH) or 10 mM GSH to simulate the extracellular or intracellular physiological environment. In PBS buffer solution, no obvious peptide absorption peak was detected within 24h, suggesting that sBBI&PDP remained stable. While in 10 mM GSH solution, a rapid release of peptides could be observed. The release rate of peptides was 30% at 2 h, and it was close to 90% at 12 h (Figure 3C). In addition, BBI labeled with FITC (Green) and PDP labeled with Cy5 (Red) were used to track the locations of peptides release from the sBBI&PDP nanoparticles in the tumor cells, which was observed densely in cytoplasm of tumor cell and faintly in nucleus (Figure S6). The fluorescence of the two peptides did not overlap closely. It was likely that the different binding sites of the two peptides of sBBI&PDP was different in tumor cells, Which BBI interacted with β-catenin/BCL9 and PDP binded to PD-L1, respectively. These results suggested that sBBI&PDP could be triggered by intracellular GSH for drug release.

The metabolizable nanoparticles are an emerging class of engineered nanomedicines that can direct payloads to target sites distinct from targeted nanoparticles, functional nanocarriers can pass through urine and/or can be systematically cleared by the mononuclear phagocyte system (MPS) 46, 47. And these nanoparticles have great potential to mitigate systemic toxicity by preventing nonspecific accumulation in healthy tissues and organs 48. To further investigate the cleanability and pharmacokinetics of the sBBI&PDP nanoparticles *in vivo*, we mass-spectrographically monitored the pharmacokinetic data of sBBI&PDP in C57BL/6J mice after tail vein injection. The ^197^Au level in the blood of healthy mice were measured using inductively coupled plasma mass spectrometry (ICP-MS). Time-dependent measurements (expressed in ng/mL) and metabolic kinetics of ^197^Au in blood showed that sBBI&PDP nanoparticles had a half-life of 8.5 hours. After 48 hours, more than 90% of ^197^Au was cleared from the blood, and almost all ^197^Au was depleted after one week (Figure 3D). Therefore, administration with once every 2 days was a safe dosage. One week after injection, hardly any ^197^Au were found in the organs included heart, lung, kidney, spleen or liver (Figure 3D). In particle, the liver and spleen took over most of the metabolic duties of ^197^Au (Figure 3D), suggested that sBBI&PDP nanoparticle was excluded from the body in an MPS-dependent manner. Taken together, these results demonstrated that sBBI&PDP was a clearable nanoparticle.

### Anti-tumor mechanism of sBBI&PDP *in vitro* and *in vivo*

**Figure 4:**
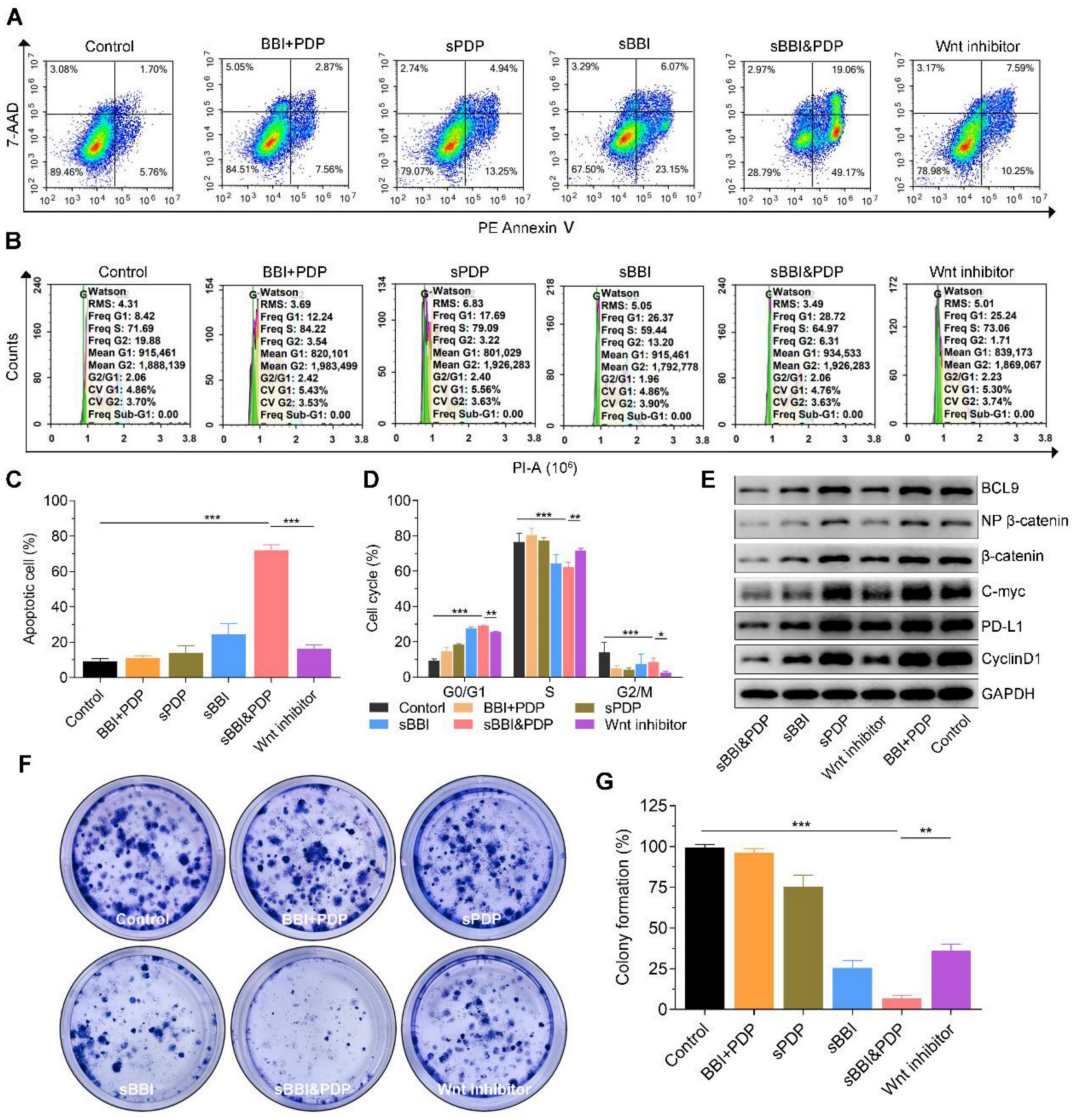
** Anti-tumor efficacy and mechanism of sBBI&PDP *in vitro*.** *In vitro* analysis of hepatocellular carcinoma cells (Hepa1-6) after exposure to 20 μM sBBI&PDP with 48 hours compared with Control (PBS), pure two peptide mixtures (BBI+PDP), sPDP, sBBI or Wnt inhibitor. (A, C) Flow cytometry analysis shown that the proportion of apoptotic cells in sBBI&PDP significantly increased than the above five groups. (B, D) Flow results of cell cycle indicated G0/G1 phase significantly increased and S phase decreased in sBBI&PDP group compared with the above five groups. (E) Western blotting shown that the protein levels of BCL9, Non-phosphorylated (active) β-catenin, β-catenin, c-Myc, PD-L1 and CyclinD1 in sBBI&PDP were significantly down-regulated compare with the above groups. (F-G) Colony formation indicated that cloned spheres in sBBI&PDP significantly were less than the above five groups (*n* = 3, *t*-test, *p < 0.001, **p < 0.001, ***p < 0.001)

To test our hypothesis, we investigated the mechanism by which sBBI&PDP recruit and sensitize CD8^+^T cells to kill hepatoma by inhibiting β-catenin and PD-L1 protein expression *in vitro* and *in vivo*. First, the images (Figure 4A) and statistical analysis (Figure 4C) of apoptosis studies *in vitro* showed that Hepa1-6 cells exposed to 20 μM sBBI&PDP after 48 hours had significantly more apoptotic cells than Hepa1-6 cells exposed to the same concentration of Wnt inhibitor, sBBI, sPDP, pure two peptide mixtures (BBI+PDP) and Control. And the images (Figure 4B) and statistical analysis (Figure 4D) of cell cycle experiments showed that compared with Wnt inhibitor, sBBI, sPDP, BBI+PDP and Control, Hepa1-6 cells treated with sBBI&PDP were significantly arrested in G0/G1 phase and downregulated in S phases. The WB experiments of Hepa1-6 cells treated with the above administration shown that the protein levels of BCL9, Non-phosphorylated (active) β-catenin, β-catenin, c-Myc, PD-L1 and CyclinD1 in sBBI&PDP group were significantly down-regulated compared with Wnt inhibitor, sBBI, sPDP, BBI+PDP and Control (Figure 4E). The previous study demonstrated that β-Catenin could regulate the expression of tumor-derived PD-L1 49. Moreover, Wnt signaling can also promote c-Myc expression and function, which also stimulates PD-L1 transcription 50. In our studies, BBI or PDP released from sBBI&PDP in tumor cells. BBI could prevented the interaction of β-catenin and BCL9 in tumor cells, inhibited the activation of the canonical Wnt/β-catenin signaling pathway and down-regulated the PD-L1 level in the tumors. Besides, PDP interacted with intramembranous segment of PD-L1 could also influence the expression of PD-L1 in the tumor cells. Additionally, Hepa1-6 cloning experiments showed that the cloning photo (Figure 4F) and formation rate (Figure 4G) exposed to *s*BBI&PDP were significantly lower than those in the Wnt inhibitor, sBBI, sPDP, BBI+PDP and Control group. Besides, cytotoxicity experiments (Figure S7) showed that the cell viability of Hepa1-6 cells treated with sBBI&PDP at concentrations of 3.125, 6.25, 12.5, 25 and 50 μM was significantly lower than that of sBBI and Control at the same concentration. The above *in vitro* experiments showed that sBBI&PDP inhibited cell proliferation and promoted cell apoptosis and necrosis by reducing the expression of β-catenin and PD-L1 proteins in tumor cells.

**Figure 5:**
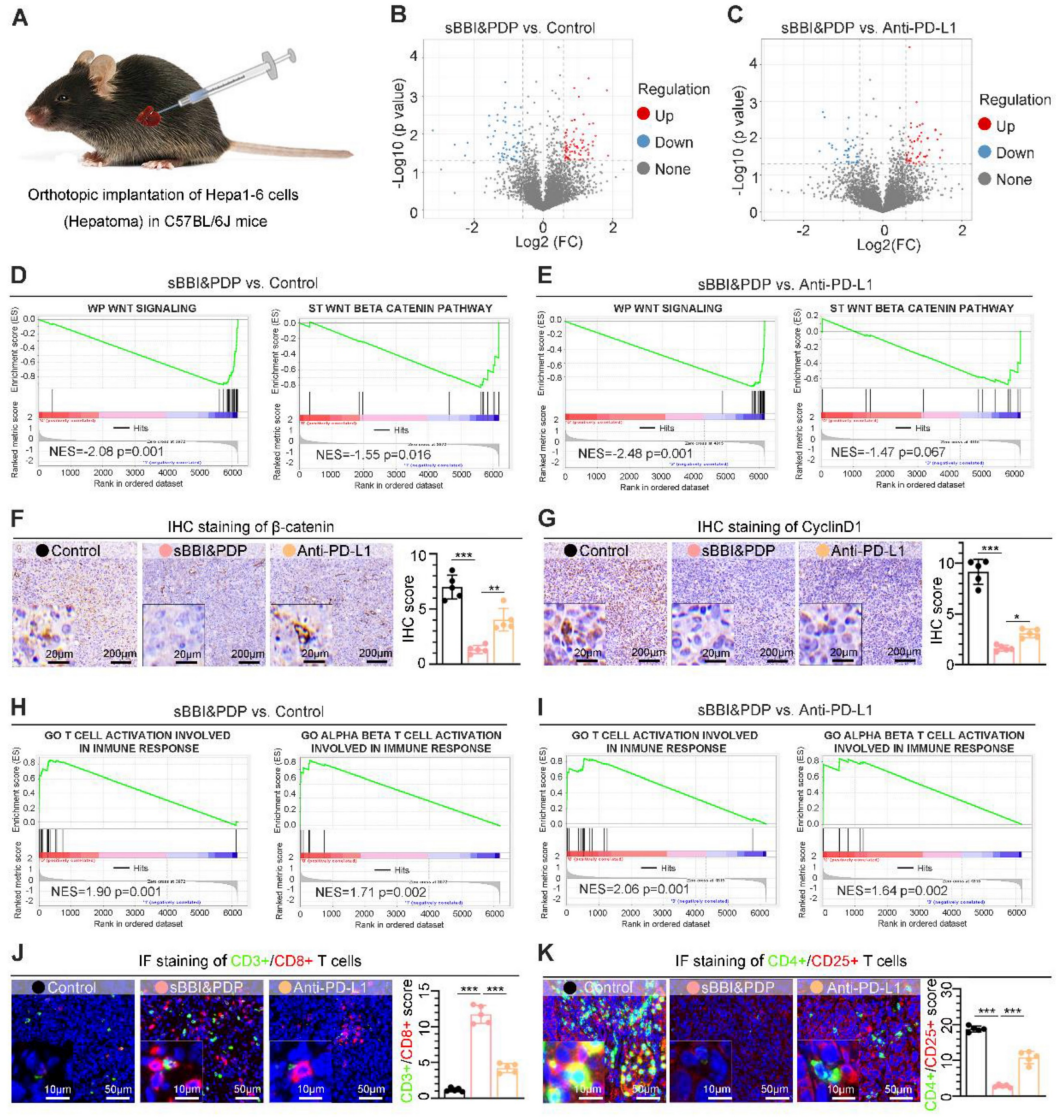
** Anti-tumor mechanism of sBBI&PDP *in vivo*.** (A) Liver orthotopic implantation model of Hepa1-6 cells in C57BL/6J mice. (B, C) Volcano plots of proteins differentially expressed in hepatoma tissues of C57 mice after administration with 3 mg/kg *s*BBI&PDP compared to Control (B) or Anti-PD-L1 (C). (D, E) Gene set enrichment analysis (GSEA) showing the WP Wnt signaling and ST Wnt beta-catenin pathway differentially expressed in response to sBBI&PDP compared with Control (D) or Anti-PD-L1 (E). NES, normalized enrichment score. (F, G) Immunohistochemical (IHC) staining analysis of representative hepatoma sections with the β-catenin (F) and CyclinD1 (G) (scare bar: 100 μm, enlarged drawing: 20 μm). (H, I) GSEA showing the GO T cell or GO alpha beta T cell activation involved in immune response differentially expressed in response to sBBI&PDP compared with Control (H) or Anti-PD-L1 (I). (J, K) Immunofluorescence (IF) staining analysis of representative hepatoma sections with the CD3^+^/CD8^+^ T cell (J) and CD4^+^/CD25^+^ T cell (K) (scare bar: 50 μm, enlarged drawing: 10 μm). (*n* = 5. *t*-test, *p < 0.05; **p < 0.01; ***p < 0.001).

To further study the antitumor mechanism of sBBI&PDP *in vivo*, we established an orthotopic HCC mouse model (Figure 5A). By proteomic (Label-free) sequencing analysis of liver cancer tissues, sBBI&PDP had a large number of differentially expressed proteins compared with Control (Figure 5B) or commercial PD-L1 inhibitor (Anti-PD-L1) (Figure 5C). Further gene set enrichment analysis (GSEA) showed that the Wnt signaling and Wnt-β/catenin pathway in the sBBI&PDP intervention group were significantly down-regulated compared with the Control group (Figure 5D) or the Anti-PD-L1 treatment group (Figure 5E). The immunohistochemical (IHC) results of corresponding liver cancer tissue sections showed that the expression levels of β-catenin (Figure 5F) and CyclinD1 (Figure 5G), the main related proteins of Wnt signaling pathway, were also significantly down-regulated in sBBI&PDP compared to Control or Anti-PD-L1, consistent with GSEA results. More importantly, the GSEA results in the sBBI&PDP group were significantly upregulated in immune response-associated T cell or αβ-T cell activation compared with the Control group (Figure 5H) or Anti-PD-L1 treatment (Figure 5I). The corresponding immunofluorescence double staining results showed that CD3^+^/CD8^+^ T cells (Figure 5J) were significantly upregulated and CD4^+^/CD25^+^ Treg cells (Figure 5K) were downregulated in sBBI and PDP groups compared with Control or Anti-PD-L1. Additionally, in the experiment of immune cell analysis by flow cytometry, compared with Control and Anti-PD-L1, sBBI&PDP can significantly increase the proportion of CD8^+^ cytotoxic T lymphocytes (CTLs) (Figure S8A) and reduce the proportion of regulatory T cells (Tregs) (Figure S8B) in tumor tissue, indicating that sBBI&PDP can improve the immune response of the body against liver cancer. To sum up, the above results show that *s*BBI&PDP recruits more CD8^+^ T cells to act on malignant liver tumor cells *in vivo*, and increases the sensitivity of CD8^+^ T cells to kill tumors of hepatocellular carcinoma by inhibiting Wnt/β-catenin and PD-L1.

### Anti-tumor therapeutic effect of sBBI&PDP in a homograft mice model of HCC

**Figure 6:**
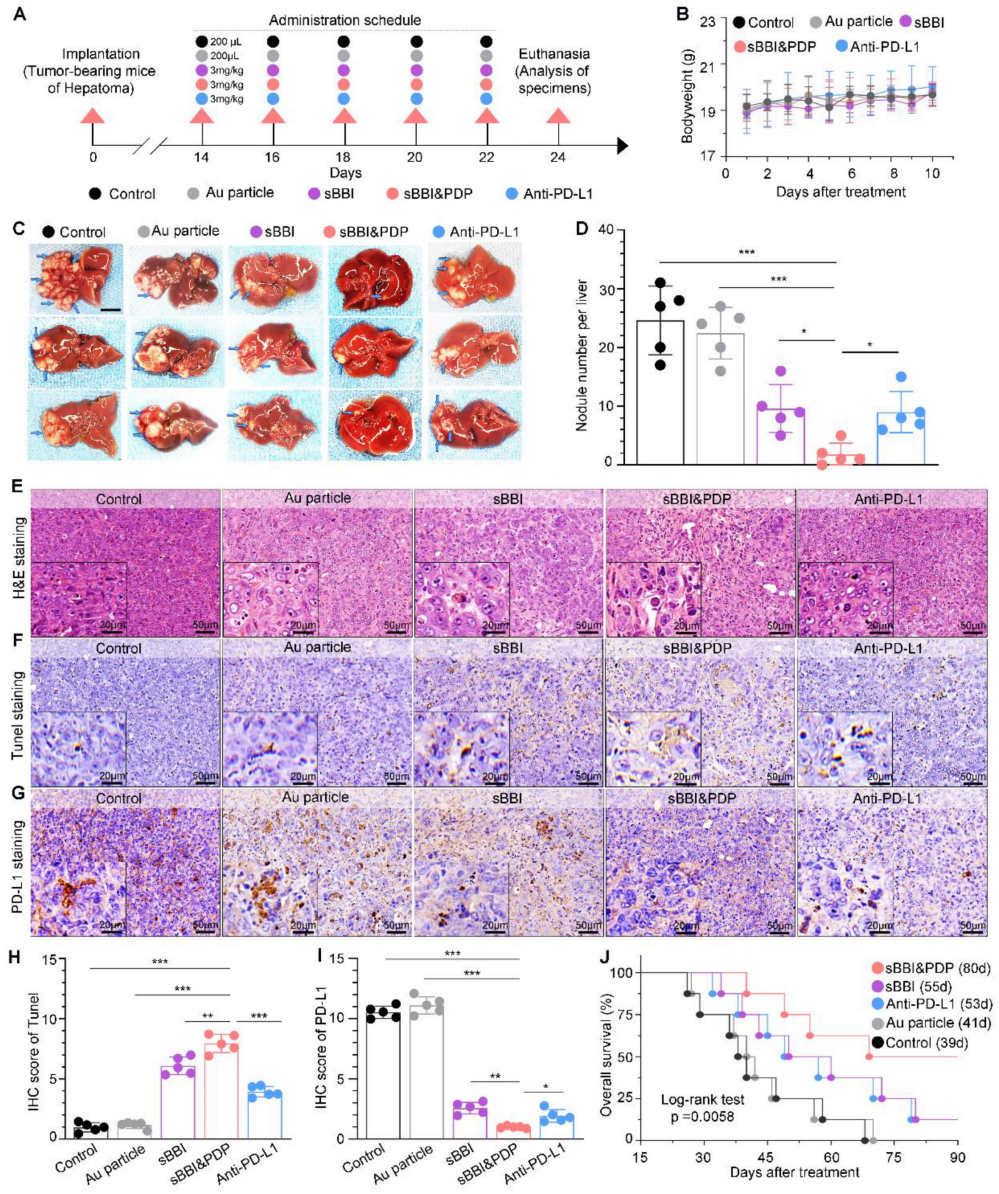
** Anti-tumor therapeutic effect of sBBI&PDP in an orthotopic homograft mice model of HCC.** (A) Scheme of the tumor-bearing mice of liver cancer form implantation, administration to analysis of specimens. They were divided into 5 groups according to the experimental plan, respectively, Control, Au particle, sBBI, sBBI&PDP and Anti-PD-L1. (B) Body weight changes of each group of mice during the administration. (C) Representative liver tumor images are shown (scare bar: 0.5 cm). Arrows indicate tumor nodules. (D) Nodule number per liver in sBBI&PDP were compared with Control, Au particle, sBBI or Anti-PD-L1 (*n* = 5. *t*-test, *p < 0.05, **p < 0.01, ***p < 0.001). (E-G) Staining analysis of the representative hepatoma tissue sections each group after indicated treatment, respectively, H&E staining (E), TUNEL staining (F) and PD-L1 staining (G) (scare bar: 50 μm, enlarged drawing: 20 μm). (H-I) IHC scores of TUNEL (H) and PD-L1 (I) in sBBI&PDP were compared with Control, Au particle, sBBI or Anti-PD-L1 (*n* = 5. *t*-test, *p < 0.05, **p < 0.01, ***p < 0.001). (J) Overall survival analysis of sBBI&PDP was compared with Control, Au particle, sBBI or Anti-PD-L1 in the 90-days trial period. The median survival time was in parentheses (Log-rank test, *p* = 0.0058).

In order to study the therapeutic effect of sBBI&PDP on HCC, we used the orthotopic homograft mice model of HCC to study its curative effect. The tumor formation time was about 2 weeks, and then randomly divided into 5 groups for administration (Figure 6A), namely sBBI& PDP, sBBI, Anti-PD-L1, Au particle and Control group. The therapeutic dosage was 3 mg/kg, 200 μL, tail vein injection, once every 2 days, and samples were obtained for analysis after 5 dosages. Body weight changes during the administration period were recorded and there were no significant differences among the 5 groups (Figure 6B). Photographs (Figure 6C and Figure S9) of liver tumors after the dosing period, comparison of the number of tumor nodules (Figure 6D) were shown for both sBBI&PDP groups significantly reduce or eliminate the number of liver cancer nodules. The liver cancer tissue sections were then stained and analyzed. Through H&E staining (Figure 6E), TUNEL staining (Figure 6F) and TUNEL score (Figure 6H), we found that sBBI&PDP had significantly more necrotic and apoptotic cells in tumor tissue than sBBI, Anti-PD-L1, Au particle or Control. IHC staining (Figure 6G) and assessment (Figure 6I) of PD-L1 protein showed that PD-L1 level were significantly downregulated in sBBI&PDP compared with the above four groups. In addition, Ki-67 nucleoprotein staining assay, which demonstrated the proliferative activity of hepatoma cells showed that the Ki-67 level of sBBI&PDP group was significantly lower than that of sBBI, Anti-PD-L1, Au particle or Control group (Figure S10A). Besides, we also performed the IHC analysis of β-catenin and CyclinD1 protein related closely Wnt pathway in hepatoma tissues, and then it indicated that the β-catenin level of sBBI&PDP group was significantly lower than that of sBBI, Anti-PD-L1, Au particle or Control group (Figure S10B), the similar result was in CyclinD1 protein (Figure S10C). Most importantly, we found by Kaplan-Meier survival analysis (Figure 6J) that tumor-bearing mice in sBBI&PDP survived longer than those in the sBBI, Anti-PD-L1, Au particle, or Control, with a median survival time of 80 days, 55 days, 53 days, 41 days and 39 days, respectively (Log-rank test, *p* = 0.0058).

In addition, we performed also that the anti-tumor efficacy of sBBI&PDP in subcutaneous HCC mouse model (Figure S11A). Five-weeks female C57BL/6J mice were implanted by 5.0×10^6^ Hepa 1-6 cells in the right ventrodorsal area of mice. The tumor formation time was about 1 week, and then randomly divided into 6 groups for administration (3 mg/kg, 200 μL, tail vein injection), namely Control, sPDP, sBBI, sBBI&PDP, Wnt inhibitor and Anti-PD-L1 (Figure S11B). During the treatment period, the tumor volume of the sBBI&PDP group had the least change, and the tumor growth inhibition (TGI) value was significantly better than that of sBBI, Anti-PD-L1, Wnt inhibitor, sPDP and Control, which were 87.7%, 77.6%, 71.6%, 69.3%, 61.6% and 0%, respectively (Figure S11C). And body weight changes were no significant differences among the above 6 groups during the administration (Figure S11D). After treatment, the sBBI&PDP group was significantly lower than the above 5 groups in terms of tumor nodule size *in vivo* (Figure S11E) and tumor nodule weight (Figure S11F).

The above results indicated that compared with original sBBI, sPDP, commercial Wnt inhibitor or Anti-PD-L1, sBBI&PDP significantly promoted apoptosis and necrosis of HCC cells, decreased PD-L1 level and tumor proliferation activity, and significantly prolonged the survival time of HCC mice.

### Anti-tumor efficacy of sBBI&PDP in the HCC PDX model of hu-PBMC-NSG mice

**Figure 7:**
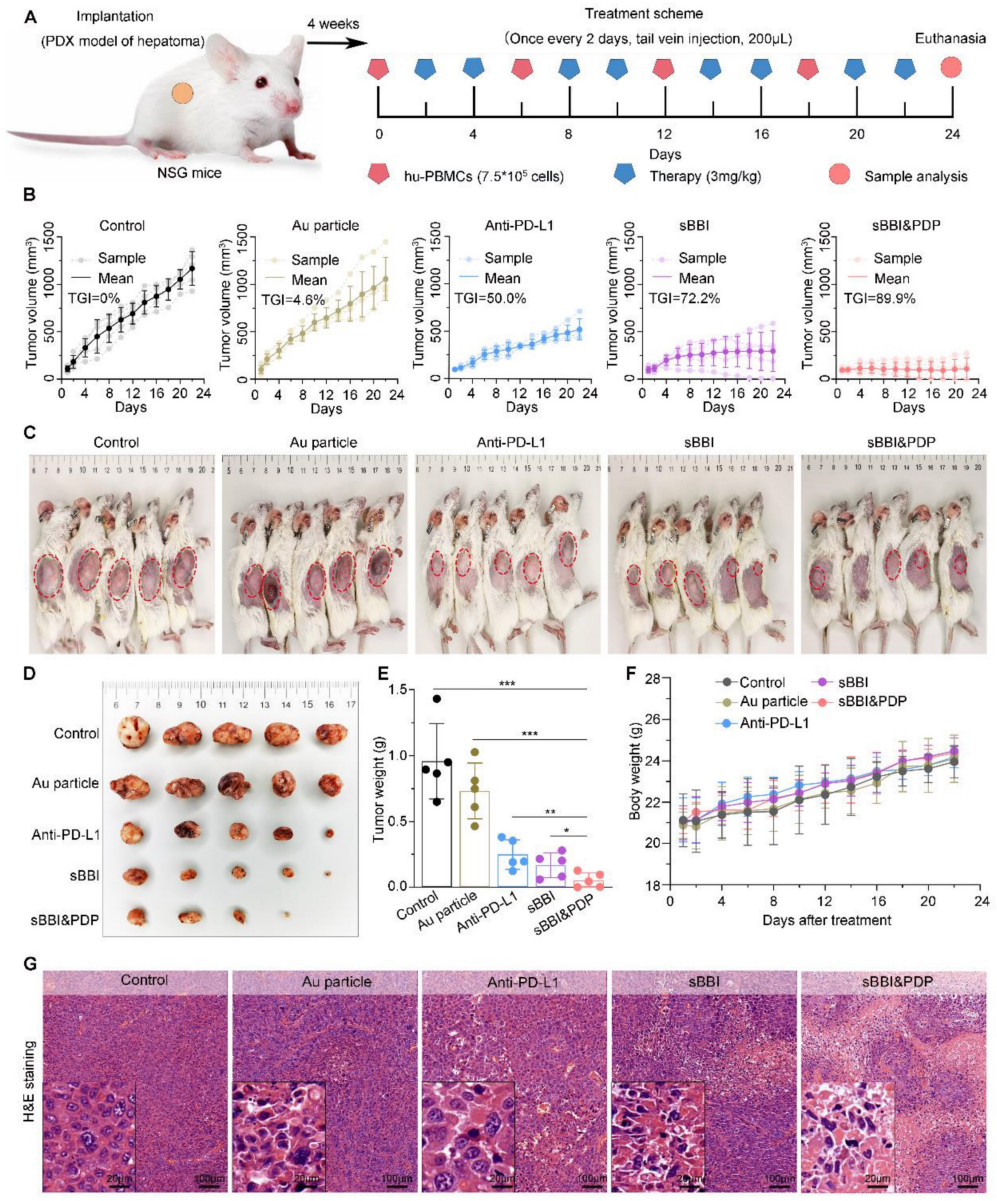
** Anti-tumor efficacy of sBBI&PDP in the PDX HCC model of hu-PBMC-NSG mice.** (A) Schematic diagram of the HCC-PDX model implantation, adoptive transplant of PBMCs, corresponding treatment and sample analysis in NSG mice. (B) Growth curves of HCC in NSG mice with the indicated treatments (TGI, tumor growth inhibition; *n* = 5/group). (C) HCC images *in vivo* were shown and red circles indicated the subcutaneous tumor nodules. (D, E) Tumor nodule images *in vitro* (D) and matching tumor weight (E) in *s*BBI&PDP were compared with Control, Au particle, sBBI or Anti-PD-L1 (*n* = 5. *t*-test, *p < 0.05, **p < 0.01, ***p < 0.001). (F) Body weight changes during the 24 days treatment. (G) H&E staining analysis of the representative hepatoma tissue sections each group after indicated treatment (scare bar: 100 μm, enlarged drawing: 20 μm).

In order to further study the therapeutic effect of sBBI&PDP for HCC, we conducted relevant experiments in the hu-PBMC-NSG mice (Figure 7A). In the HCC PDX model, tumors developed within 4 weeks, and then they were divided into 5 treatment groups: sBBI&PDP, sBBI, Anti-PD-L1, Au particle and Control. Prior to treatment (materials 3 mg/kg, 200 μL, tail vein injection), peripheral blood mononuclear cells (PBMCs) (7.5×10^5^ cells per mouse, 200 μL, intravenous injection, once every 6 days) were administered to reconstitute the human immune system (Figure 7A). Changes in tumor volume and body weight of mice were recorded. During the treatment period, the tumor volume of the sBBI&PDP group changed less, and the TGI value was significantly better than that of sBBI, Anti-PD-L1, Au particle and Control, which were 89.9%, 72.2%, 50.0%, 4.6% and 0%, respectively (Figure 7B). After treatment, the sBBI&PDP group was significantly lower than the above four groups in terms of tumor nodule size *in vivo* (Figure 7C), tumor nodule size *in vitro* (Figure 7D) and tumor nodule weight (Figure 7E). In addition, there was no significant difference in body weight changes of the five groups of tumor-bearing mice during the administration period (Figure 7F). The results show that sBBI&PDP is safe and effective in the treatment of liver cancer.

**Figure 8:**
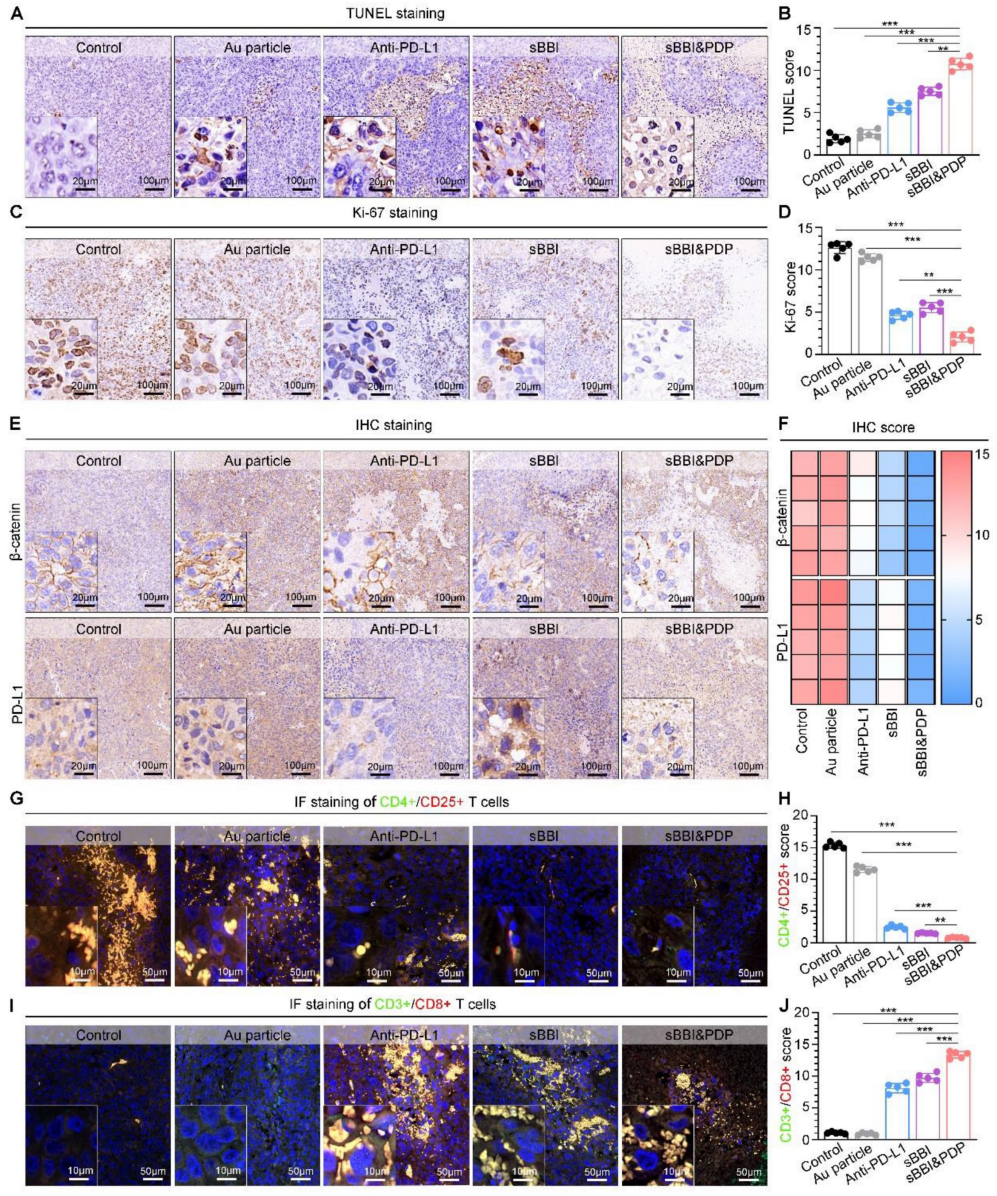
** Anti-tumor mechanisms of sBBI&PDP in the HCC-PDX model.** (A, B) TUNEL staining (A) and scores (B) of representative HCC tissue sections were differentially expressed in sBBI&PDP group compared with Control, Au particle, sBBI or Anti-PD-L1 (scare bar: 100 μm, enlarged drawing: 20 μm. *n* = 5, *t*-test, **p < 0.01, ***p < 0.001). (C, D) Ki-67 staining (C) and scores (D) of representative HCC tissue sections were differentially expressed in sBBI&PDP group compared with Control, Au particle, *s*BBI or Anti-PD-L1 (scare bar: 100 μm, enlarged drawing: 20 μm. *n* = 5, *t*-test, **p < 0.01, ***p < 0.001). (E, F) IHC staining (E) and scores of heat map (F) of β-catenin or PD-L1 protein of representative HCC tissue sections were differentially expressed in *s*BBI&PDP group compared with Control, Au particle, sBBI or Anti-PD-L1 (scare bar: 100 μm, enlarged drawing: 20 μm. *n* = 5). (G, H) CD4^+^/CD25^+^ T cell staining (G) and scores (H) of representative HCC tissue sections were differentially expressed in sBBI&PDP group compared with Control, Au particle, *s*BBI or Anti-PD-L1. (I, J) CD3^+^/CD8^+^ T cell staining (I) and scores (G) of representative HCC tissue sections were differentially expressed in sBBI&PDP group compared with Control, Au particle, sBBI or Anti-PD-L1 (scare bar: 50 μm, enlarged drawing: 10 μm. *n* = 5, *t*-test, **p < 0.01, ***p < 0.001).

To investigate the mechanism of sBBI&PDP treating liver cancer in the PDX model, we performed staining analysis on liver cancer tissues. H&E staining of liver tumors (Figure 7G) showed that the sBBI&PDP group had a higher degree of tumor tissue necrosis and sparse cells, and the sBBI and Anti-PD-L1 group also had a certain degree of necrosis. In contrast, the tumor tissues of the Control and Au particle group were relatively complete and the cells were dense, with large and dark nucleus. This was also confirmed by TUNEL analysis, with more apoptotic and dead cells in the sBBI&PDP group compared with sBBI, Anti-PD-L1, Au particle and Control group (Figure 8A-B). Tumor cell nuclear protein Ki-67 staining (Figure 8C) and evaluation (Figure 8D) showed that the Ki-67 level in the sBBI&PDP group was significantly lower than that in the sBBI, Anti-PD-L1, Au particle or Control group. Then we performed immunohistochemical staining (Figure 8E) and heat map scoring (Figure 8F) on β-catenin and PD-L1 proteins, and found that the β-catenin and PD-L1 protein levels in the sBBI&PDP group were significantly lower than those in the sBBI, Anti-PD-L1, Au particle or Control group. Finally, immunofluorescence staining analysis was performed on liver cancer tissues, and it was found that the infiltration of CD4^+^/CD25^+^ T cells (Figure 8G-H) in the sBBI&PDP group was lower than that of the above four groups, but the infiltration of CD3^+^/CD8^+^ T cells (Figure 8I-J) was significantly increased. Fewer tumor cells in hepatocellular carcinoma tissue sections. In brief, these results indicated that sBBI&PDP inhibited the expression of β-catenin and PD-L1 protein by targeting liver cancer, activating immune response and killing liver cancer cells in HCC-PDX model. Its therapeutic effect for HCC is significantly better than sBBI and Anti-PD-L1, and it has certain safety.

### Biocompatibility of sBBI&PDP

**Figure 9:**
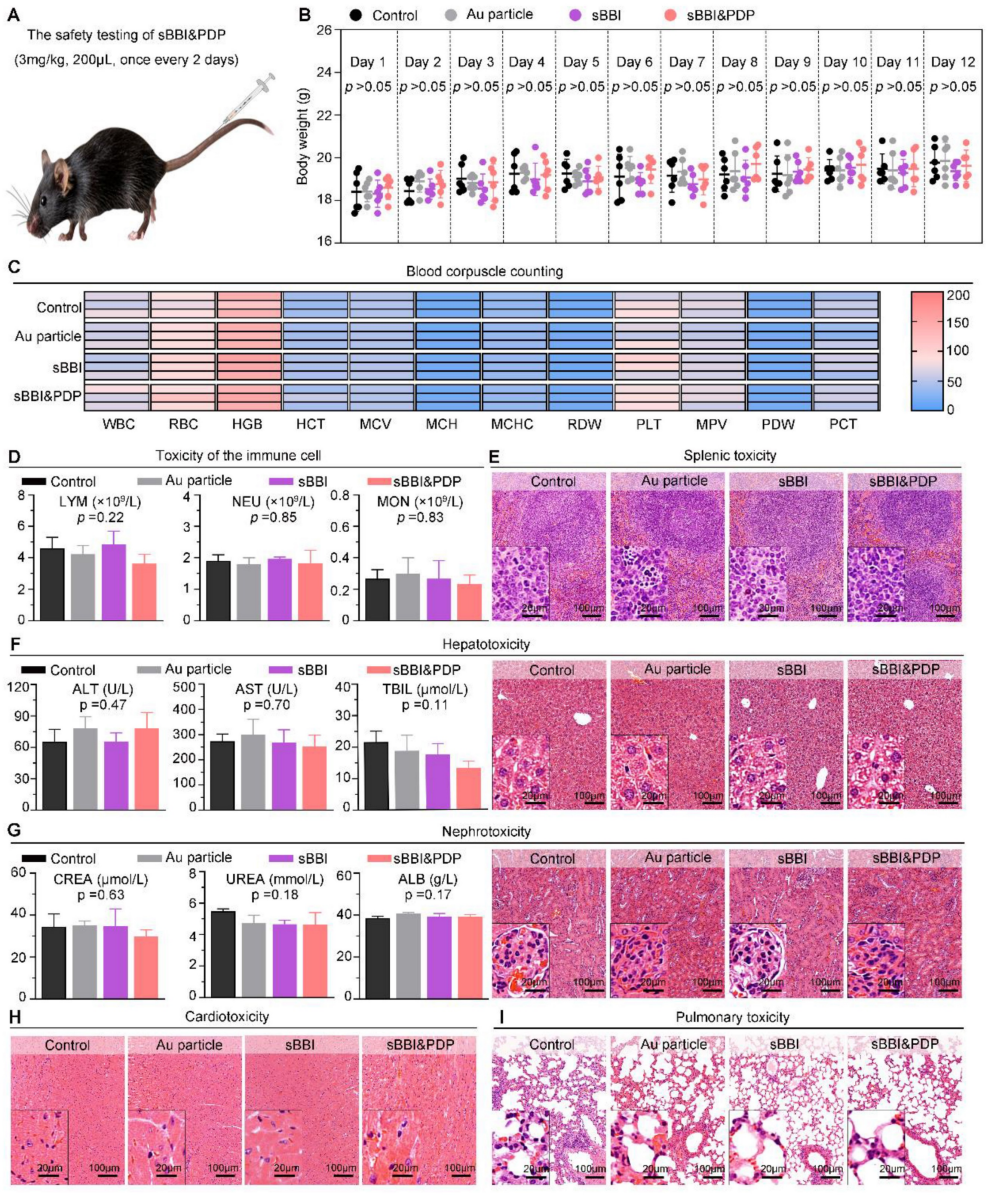
** Biocompatibility of sBBI&PDP.** (A) Administration scheme of safety testing *in vivo*. (B) Body weight changes of mice after exposure to 3 mg/kg sBBI&PDP during the 12-days administration compared with Control, Au particle or sBBI (*n* = 6/group). (C) The heat map of blood corpuscle counting in mice upon different treatments at the end of the experiment (*n* = 3/group). (D) Toxicity analysis of the immune cells included lymphocyte (LYM), neutrophil (NEU) and monocyte (MON) after indicated treatment (*n* = 3/group). (E) Splenic toxicity with hematoxylin and eosin (H&E) staining of the above different groups. (F) Hepatotoxicity with the H&E staining and serum biochemical indexes included alanine aminotransferase (ALT), aspartate aminotransferase (AST) and total bilirubin (TBIL) after indicated treatments. (G) Nephrotoxicity with the H&E staining and serum biochemical indexes included creatinine (CREA), urea and albumin (ALB) after indicated treatments. (H, I) Cardiotoxicity (H) and Pulmonary toxicity (I) with the H&E staining of the above different groups. Scare bar: 100 μm, enlarged drawing: 20 μm. Statistics analysis was performed using one-way analysis of variance.

In order to further investigate the biological safety of sBBI&PDP, we conducted an *in vivo* experiment-C57 mouse model (Figure 9A). The administration of materials was 3 mg/kg, 200 μL, tail vein injection, once every 2 days, and samples were obtained for analysis after a total of 5 dosing cycles. In the 12-day dosing model, there was no significant changes of mouse body weight in the sBBI&PDP group compared with the sBBI, Au particle or Control group (Normal saline) (Figure 9B). Next, comparing the blood count heatmaps of routine hematology at the end point of the *in vivo* safety experiment (Figure 9C), we concluded that there was no significant difference in the effects of the above four groups on routine hematology in mice. Immunocytotoxicity analysis (Figure 9D) showed no statistical differences between the four groups in terms of adaptive immune cells, lymphocytes (LYM) and innate immune cells, neutrophils (NEU) and monocytes (MON). And then, the biocompatibility of sBBI&PDP with different organs was further examined. The H&E staining of representative splenic tissue sections and partial magnification of splenic white pulp showed no significant difference in splenic toxicity between the four groups (Figure 9E). Hepatotoxicity was observed by indicators of liver function (alanine aminotransferase (ALT), aspartate aminotransferase (AST), total bilirubin (TBIL)), H&E staining of representative liver tissue sections, and partial enlargement of liver parenchyma (Figure 9F). There was no statistical difference between the groups. Liver MASSON or H&E staining (Figure S12) also showed no obvious fibrosis or necrotic cells in the liver parenchyma in the four groups. Nephrotoxicity using markers of renal function, serum creatinine (CREA), UREA, and albumin (ALB) (Figure 9G), and H&E staining of representative liver tissue sections and glomerular enlargements illustrate no significant differences between the groups. Cardiopulmonary toxicity was also not significantly different between groups, with H&E staining in representative sections of heart tissue and myocardial fibers (Figure 9H) and lung tissue and alveolus (Figure 9I). The above results indicated that sBBI&PDP exhibited good multi-organ biocompatibility at therapeutic doses without obvious toxicity.

## Conclusion

Protein-protein interactions (PPIs) regulate and control almost all biological processes, including tumorigenesis, and the targeting of PPIs has become a viable tumor treatment strategy in recent years 51. Extensive biological and clinical studies have identified centers and nodes of oncoprotein interactions that are critical for the acquisition and maintenance of cancer properties required for cellular transformation. Oncogenic PPIs have emerged as promising therapeutic targets. The discovery of PPI modulators and the verification of PPI-targeted drugs in clinical trials have made PPI-targeted anti-cancer strategies a reality 52. There is an urgent need to develop PPI-based personalized anticancer drugs to improve the treatment outcomes of cancer patients.

To addressed it, in this work, a racemic spherical supramolecular peptide termed sBBI&PDP was constructed by hierarchical self-assembly, comprising an L-enantiomeric peptide as an inhibitor of BCL9 and catenin (sBBI) and a D-enantiomeric peptide as an inhibitor of PD-1/PD-L1 (PDP) (Figure 1). As expected, sBBI&PDP potently suppressed the hyperactivated Wnt/β-catenin pathway *in vitro* and *in vivo*, while blocking endogenous PD-L1 effectively. BBI prevented the interaction of β-catenin and BCL9 in tumor cells, inhibited the activation of the canonical Wnt/β-catenin signaling pathway, thereby inhibiting the growth of tumor cells and recruiting more CD8+ T cells to act on the tumor (Figure 1). On the other hand, PDP binds to PD-L1 molecules on the surface of tumor cells, blocks the interaction of PD-1/PD-L1, sensitizes drug-resistant CD8+ T cells, and enhances the effect of killing tumor cells (Figure 1). Notably, compared with the original sBBI and commercial Anti-PD-L1 inhibitors, the designed sBBI&PDP showed stronger antitumor efficacy in an orthotopic homograft mice model of HCC and a PDX HCC model in hu-PBMC-NSG mice. Moreover, sBBI&PDP possessed a favorable biosafety profile. Collectively, the successful implementation of this strategy could revitalize ICB blockage therapy and promote the discovery of artificial peptides for HCC therapy.

## Supplementary Material

Supplementary figures and methods.

## References

[1] Villanueva A (2019). Hepatocellular Carcinoma. N Engl J Med.

[2] Llovet JM, De Baere T, Kulik L, Haber PK, Greten TF, Meyer T (2021). Locoregional therapies in the era of molecular and immune treatments for hepatocellular carcinoma. Nat Rev Gastro Hepat.

[3] Sangro B, Sarobe P, Hervas-Stubbs S, Melero I (2021). Advances in immunotherapy for hepatocellular carcinoma. Nat Rev Gastro Hepat.

[4] Zhang Y, Wang X (2020). Targeting the Wnt/β-catenin signaling pathway in cancer. J Hematol Oncol.

[5] Ruiz de Galarreta M, Bresnahan E, Molina-Sanchez P, Lindblad KE, Maier B, Sia D (2019). Beta-catenin activation promotes immune escape and resistance to anti-PD-1 therapy in hepatocellular carcinoma. Cancer Discov.

[6] Spranger S, Bao R, Gajewski TF (2015). Melanoma-intrinsic beta-catenin signalling prevents anti-tumour immunity. Nature.

[7] Sharma P, Hu-Lieskovan S, Wargo JA, Ribas A (2017). Primary, adaptive, and acquired resistance to cancer immunotherapy. Cell.

[8] Takeuchi Y, Tanegashima T, Sato E, Irie T, Sai A, Itahashi K (2021). Highly immunogenic cancer cells require activation of the Wnt pathway for immunological escape. Sci Immunol.

[9] Wang B, Tian T, Kalland KH, Ke X, Qu Y (2018). Targeting Wnt/β-catenin signaling for cancer immunotherapy. Trends Pharmacol Sci.

[10] Liu T, Yan J, He C, You W, Ma F, Chang Z (2021). A tumor-targeting metal-organic nanoparticle constructed by dynamic combinatorial chemistry toward accurately redressing carcinogenic Wnt cascade. Small.

[11] Berraondo P, Ochoa MC, Olivera I, Melero I (2019). Immune desertic landscapes in hepatocellular carcinoma shaped by β-catenin activation. Cancer Discov.

[12] Ma F, Liu T, Yang W, You W, He W, Yan J (2022). Turning fluvastatin into a supramolecular immuno-sensitizer towards augmented tumor immunotherapy. Chem Eng J.

[13] Liu J, Xiao Q, Xiao J, Niu C, Li Y, Zhang X (2022). Wnt/β-catenin signalling: function, biological mechanisms, and therapeutic opportunities. Signal Transduct Tar.

[14] Takada K, Zhu D, Bird GH, Sukhdeo K, Zhao JJ, Mani M (2012). Targeted disruption of the BCL9/β-catenin complex inhibits oncogenic Wnt signaling. Sci Transl Med.

[15] Jiang M, Kang Y, Sewastianik T, Wang J, Tanton H, Alder K (2020). BCL9 provides multi-cellular communication properties in colorectal cancer by interacting with paraspeckle proteins. Nat Commun.

[16] Feng M, Wu Z, Zhou Y, Wei Z, Tian E, Mei S (2021). BCL9 regulates CD226 and CD96 checkpoints in CD8^+^ T cells to improve PD-1 response in cancer. Signal Transduct Tar.

[17] Feng M, Jin JQ, Xia L, Xiao T, Mei S, Wang X (2019). Pharmacological inhibition of β-catenin/BCL9 interaction overcomes resistance to immune checkpoint blockades by modulating Treg cells. Sci Adv.

[18] Jubb H, Higueruelo AP, Winter A, Blundell TL (2012). Structural biology and drug discovery for protein-protein interactions. Trends Pharmacol Sci.

[19] Sikandar A, Koehnke J (2019). The role of protein-protein interactions in the biosynthesis of ribosomally synthesized and post-translationally modified peptides. Nat Prod Rep.

[20] Buyanova M, Pei D (2022). Targeting intracellular protein-protein interactions with macrocyclic peptides. Trends Pharmacol Sci.

[21] Yin L, Cheng J, Deming TJ, Vicent MJ (2021). Synthetic polypeptides for drug and gene delivery, and tissue engineering. Adv Drug Del Rev.

[22] Song Z, Han Z, Lv S, Chen C, Chen L, Yin L (2017). Synthetic polypeptides: from polymer design to supramolecular assembly and biomedical application. Chem Soc Rev.

[23] She J, Li Y, Yan S, Yan Y, Liu D, Li S (2020). De novo supraparticle construction by a self-assembled Janus cyclopeptide to tame hydrophilic microRNA and hydrophobic molecule for anti-tumor cocktail therapy and augmented immunity. Chem Eng J.

[24] He W, Wang S, Yan J, Qu Y, Jin L, Sui F (2019). Self-assembly of therapeutic peptide into stimuli-responsive clustered nanohybrids for cancer-targeted therapy. Adv Funct Mater.

[25] Yan J, Zheng X, You W, He W, Xu GK (2022). A bionic-homodimerization strategy for optimizing modulators of protein-protein interactions: from statistical mechanics theory to potential clinical translation. Adv Sci.

[26] He W, Yan J, Sui F, Wang S, Su X, Qu Y (2018). Turning a Luffa protein into a self-assembled biodegradable nanoplatform for multitargeted cancer therapy. ACS Nano.

[27] Liu J, Yan J, Yan S, Wang Y, Zhang R, Hou P (2019). Biomimetic and self-assembled nanoclusters targeting β-catenin for potent anticancer therapy and enhanced immunotherapy. Nano Lett.

[28] Yan J, He W, Li X, You W, Liu X, Lin S (2021). Carnosic acid-induced co-self-assembly of metal-peptide complexes into a nanocluster-based framework with tumor-specific accumulation for augmented immunotherapy. Chem Eng J.

[29] Yan J, Yao Y, Yan S, Gao R, Lu W, He W (2020). Chiral protein supraparticles for tumor suppression and synergistic immunotherapy: an enabling strategy for bioactive supramolecular chirality construction. Nano Lett.

[30] Yan J, Yan S, Hou P, Lu W, Ma PX, He W (2019). A hierarchical peptide-lanthanide framework to accurately redress intracellular carcinogenic protein-protein interaction. Nano Lett.

[31] Yang G, Zhang J, Yan J, You W, Hou P, He W (2019). Modulating protein-protein interactions *in vivo* via peptide-lanthanide-ferived nanoparticles for hazard-free cancer therapy. J Biomed Nanotechnol.

[32] He W, Mazzuca P, Yuan W, Varney K, Bugatti A, Cagnotto A (2019). Identification of amino acid residues critical for the B cell growth-promoting activity of HIV-1 matrix protein p17 variants. BBA-Gen Subjects.

[33] Bian Z, Yan J, Wang S, Li Y, Guo Y, Ma B (2018). Awakening p53 by D-peptides-functionalized ultra-small nanoparticles: overcoming biological barriers to D-peptide drug delivery. Theranostics.

[34] Li L, He W, You W, Yan J, Liu W (2022). Turing miRNA into infinite coordination supermolecule: a general and enabling nanoengineering strategy for resurrecting nuclear acid therapeutics. J Nanobiotechnol.

[35] He W, Zhang Z, Yang W, Zheng X, You W, Yao Y (2022). Turing milk into pro-apoptotic oral nanotherapeutic: De novo bionic chiral-peptide supramolecule for cancer targeted and immunological therapy. Theranostics.

[36] Yang W, Liu W, Li X, Yan J, He W (2023). Turning chiral peptides into a racemic supraparticle to induce the self-degradation of MDM2. J Adv Res.

[37] Yan S, Yan J, Liu D, Li X, Kang Q, You W (2021). A nano-predator of pathological MDMX construct by clearable supramolecular gold(I)-thiol-peptide complexes achieves safe and potent anti-tumor activity. Theranostics.

[38] He W, Yan J, Li Y, Yan S, Wang S, Hou P (2020). Resurrecting a p53 peptide activator - An enabling nanoengineering strategy for peptide therapeutics. J Control Release.

[39] Yan J, Ji F, Yan S, You W, He W (2020). A general-purpose nanohybrid fabricated by polymeric Au(I)-peptide precursor to wake the function of peptide therapeutics. Theranostics.

[40] Mirzadeh N, Privér SH, Blake AJ, Schmidbaur H, Bhargava SK (2020). Innovative molecular design strategies in materials science following the aurophilicity concept. Chem Rev.

[41] Schmidbaur H, Schier A (2008). A briefing on aurophilicity. Chem Soc Rev.

[42] Zhao Y, Zhou F, Zhou H, Su H (2013). The structural and bonding evolution in cysteine-gold cluster complexes. Phys Chem Chem Phys.

[43] Tian Y, Gao Z, Wang N, Hu M, Ju Y, Li Q (2022). Engineering poly (ethylene glycol) nanoparticles for accelerated blood clearance inhibition and targeted drug delivery. J Am Chem Soc.

[44] Kennedy L, Sandhu JK, Harper ME, Cuperlovic-Culf M (2020). Role of glutathione in cancer: from mechanisms to therapies. Biomolecules.

[45] Ock K, Jeon WI, Ganbold EO, Kim M, Park J, Seo JH (2012). Real-time monitoring of glutathione-triggered thiopurine anticancer drug release in live cells investigated by surface-enhanced Raman scattering. Anal Chem.

[46] Peng C, Yu M, Hsieh JT, Kapur P, Zheng J (2019). Correlating anticancer drug delivery efficiency with vascular permeability of renal clearable versus non-renal clearable nanocarriers. Angew Chem Int Ed.

[47] Wei Q, Chen Y, Ma X, Ji J, Qiao Y, Zhou B (2018). High-efficient clearable nanoparticles for multi-modal imaging and image-guided cancer therapy. Adv Funct Mater.

[48] Kang H, Gravier J, Bao K, Wada H, Lee JH, Baek Y (2016). Renal Clearable organic nanocarriers for bioimaging and drug delivery. Adv Mater.

[49] Han C, Fu YX (2020). β-Catenin regulates tumor-derived PD-L1. J Exp Med.

[50] Casey SC, Tong L, Li Y, Do R, Walz S, Fitzgerald KN (2016). MYC regulates the antitumor immune response through CD47 and PD-L1. Science.

[51] Ivanov AA, Khuri FR, Fu H (2013). Targeting protein-protein interactions as an anticancer strategy. Trends Pharmacol Sci.

[52] Lu H, Zhou Q, He J, Jiang Z, Peng C, Tong R (2020). Recent advances in the development of protein-protein interactions modulators: mechanisms and clinical trials. Signal Transduct Tar.

