# Supplementary material for: Targeting β-catenin and PD-L1 simultaneously by a racemic supramolecular peptide for the potent immunotherapy of hepatocellular carcinoma
Source: Theranostics. 2023 Jun 4;13(10):3371–86. doi: 10.7150/thno.83377 (PMC10283047; doi:10.7150/thno.83377)
Supplement: Supplementary file 1 — Supplementary figures and methods. [file thnov13p3371s1.pdf]

Supplementary

Targeting β-catenin and PD-L1 simultaneously by a racemic Supramolecular peptide for the potent immunotherapy of Hepatocellular carcinoma

Zhengjun Zhou[1,2,†], Xiao Li[1,†], Guang Yang[3,†,*], Jingmei Wang[4], Yinong Huang[5,*], Jin Yan[2,*] Kaishan Tao[1,*]

[1]Department of Hepatobiliary and Pancreaticosplenic Surgery, Xijing Hospital, Air Force Medical University (The Fourth Military Medical University), Xi'an 710032, China;

[2]National & Local Joint Engineering Research Center of Biodiagnosis and Biotherapy, The Second Affiliated Hospital of Xi'an Jiaotong University, Xi'an, 710004, PR. China.

[3]Department of Oncology, Suzhou BenQ Medical Center, The Affiliated BenQ Hospital of Nanjing Medical University,Suzhou, Jiangsu Province China

[4]Institute for Stem Cell & Regenerative Medicine, The Second Affiliated Hospital of Xi'an Jiaotong University, Xi'an 710004, China

[5]Shaanxi Institute of Pediatric Diseases, Xi'an Children's Hospital, Xi'an, Shaanxi 710003, PR. China.

[†] These authors contributed equally.
[*] Corresponding authors:
Email: yangguang9002@163.com (G. Yang)
Email: ynhuang@xjtu.edu.cn (Y. Huang)
Email: yanjin19920602@xjtu.edu.cn (J. Yan)
Email: taokaishan0686@163.com (K. Tao)

1. Supplementary Figures

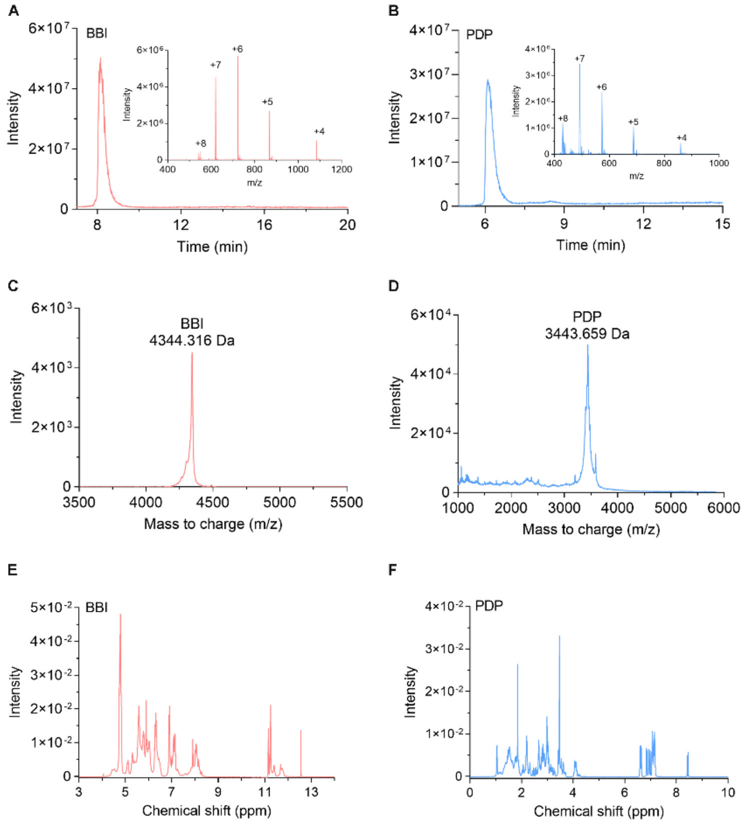

Figure S1. Characterization of synthesized BBI and PDP. (A&B) the LC-MS data of BBI (A) and PDP (B). (C&D) the HRMS data of BBI (C) and PDP (D). (E&F) 1H-NMR data of BBI (E) and PDP (F).

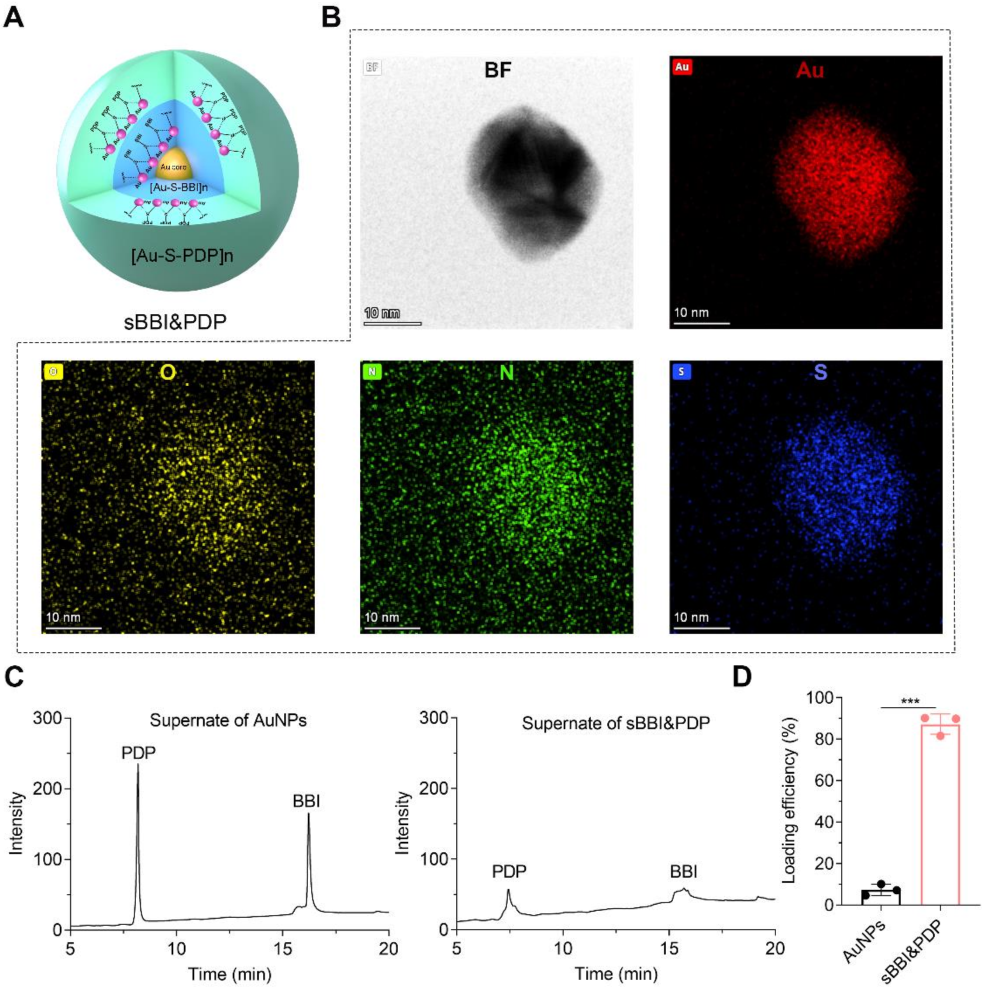

Figure S2. Properties and loading efficiency of sBBI&PDP. (A) Structure diagram of sBBI&PDP. (B) Elemental analysis included Au, O, N, S in sBBI&PDP by energy-dispersive spectroscopy (EDS) analysis. (C) HPLC detection of the uncombined BBI or PDP peptides in the supernate of AuNPs or sBBI&PDP solution. (D) Loading efficiency of BBI or PDP peptides in the sBBI&PDP was significantly higher than the AuNPs (***p<0.001).

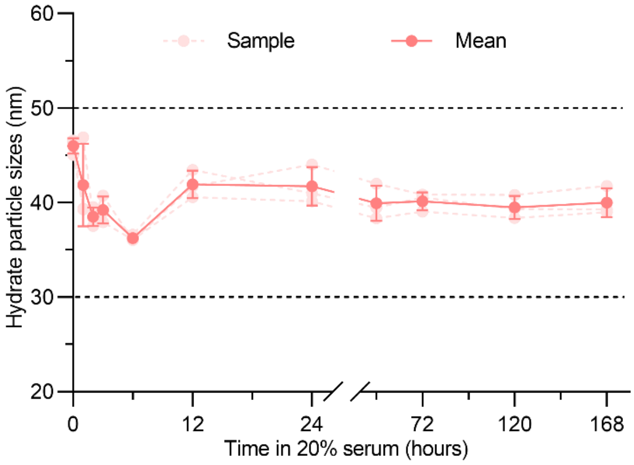

Figure S3. The colloid stability of sBBI&PDP was observed in the PBS solution containing 20% FBS by monitoring its hydrodynamic sizes.

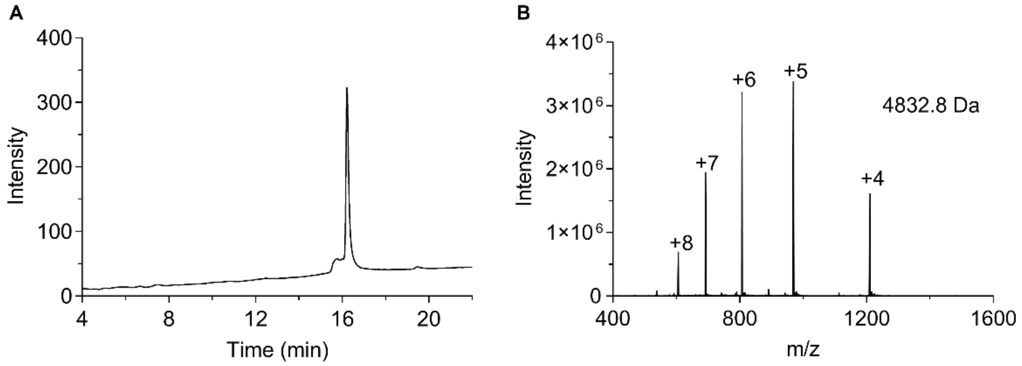

Figure S3. The characterization data of FITC-BBI. (A) HPLC and (B) Mass of FITC-BBI

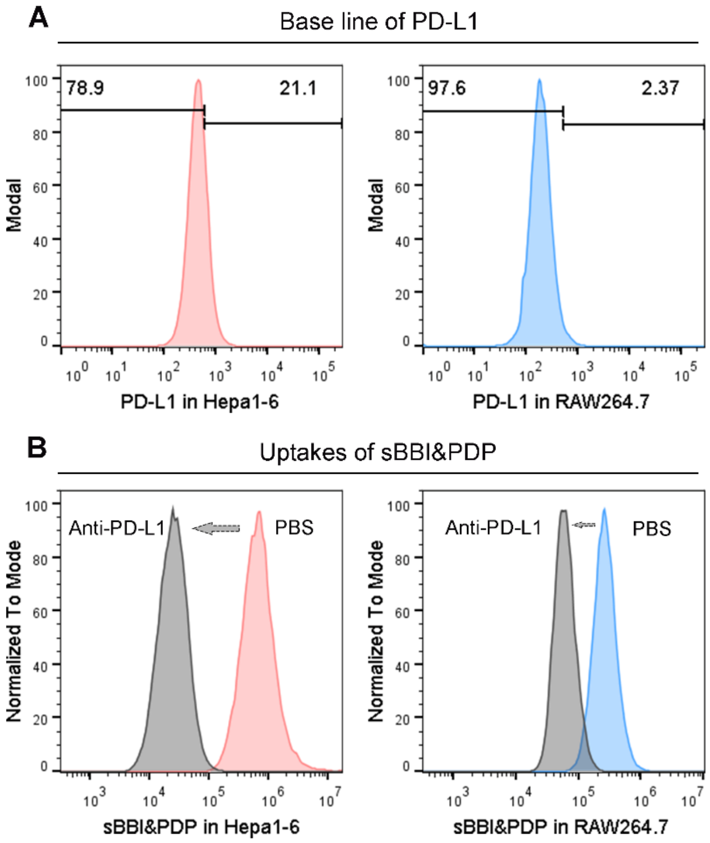

Figure S4. Flow cytometry analysis of Hepa1-6 cells and RAW264.7 macrophagocytes. (A) Base line of PD-L1 protein in Hepa1-6 cells (21.1%) was higher than RAW246.7 cells (2.37%). (B) Changes in the uptakes of sBBI&PDP after blocking cellular PD-L1 antigenic epitope of Hepa1-6 or RAW264.7 cells with PD-L1 antibody.

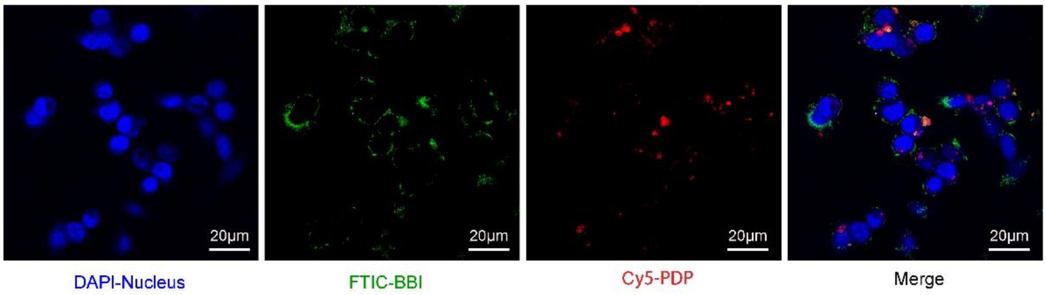

Figure S6. BBI labeled with FITC (Green) or PDP labeled with Cy5 (Red) was observed in the tumor cells.

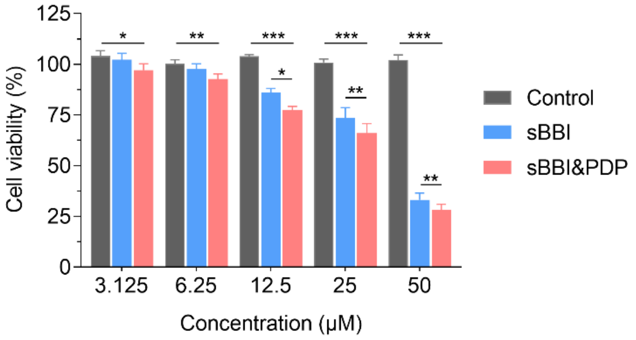

Figure S7. Cell viability of Hepa1-6 cells after exposure to 3.125, 6.25, 12.5, 25, 50μM, *s*BBI&PDP compared with Control or *s*BBI of the corresponding concentration. The statistics was used in *t*-test, $n = 3$, $*p < 0.05$, $**p < 0.01$, $***p < 0.001$.

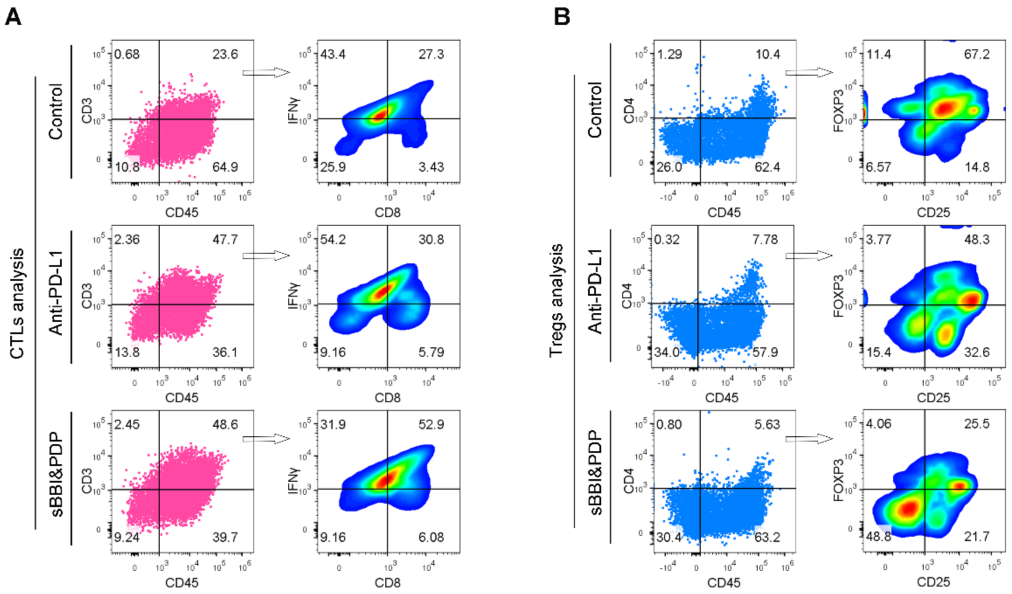

Figure S8. Flow cytometry analysis of CTLs and Tregs in HCC model treated with sBBI&PDP. (A) CTLs analysis shown that the proportion of CD45+/CD3+/CD8+/IFNγ+ T cells significantly increased in sBBI&PDP compare with Control or Anti-PD-L1 group. (B) Tregs analysis shown that the proportion of CD45+/CD4+/CD25+/FOXP+ T cells significantly decreased in sBBI&PDP compare with Control or Anti-PD-L1 group.

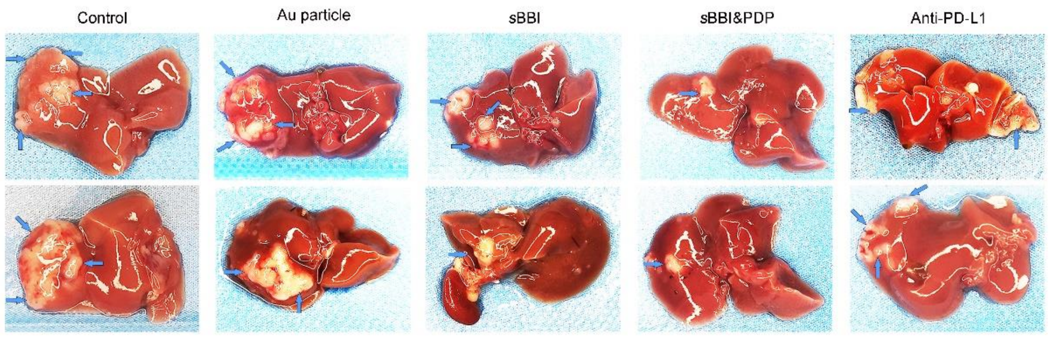

Figure S9. Anti-tumor efficacy of *s*BBI&PDP in the orthotopic hepatoma of C57 mice model. Supplementary liver images are shown, arrows indicate tumor nodules.

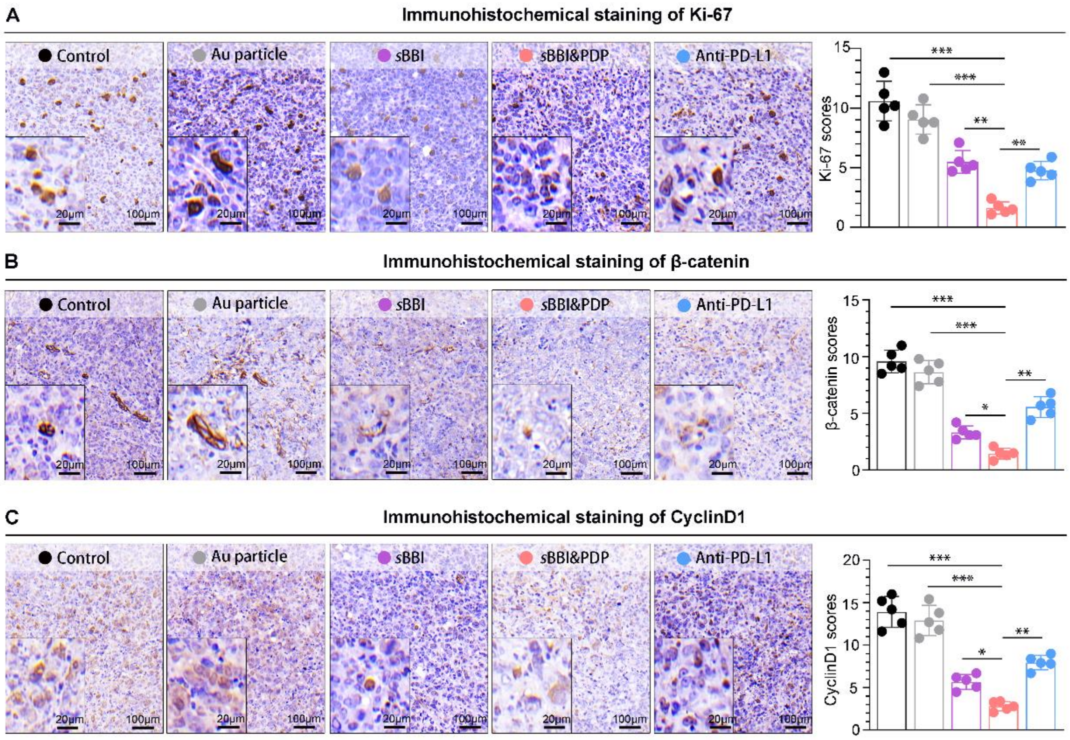

Figure S10. Anti-tumor mechanism of *s*BBI&PDP in the orthotopic hepatoma of C57 mice model. (A) TUNEL staining and scores of representative HCC tissue sections were differentially expressed in *s*BBI&PDP group compared with Control, Au particle, *s*BBI or Anti-PD-L1. (B,C) The IHC detection of proteins related to Wnt pathway in hepatoma tissue. (B) β-catenin or (C) CyclinD1 staining and scores of representative HCC tissue sections were differentially expressed in sBBI&PDP group compared with Control, Au particle, sBBI or Anti-PD-L1 (scare bar: 100μm, enlarged drawing: 20μm. $n = 5$, *t*-test, $^{**}p < 0.01$, $^{***}p < 0.001$).

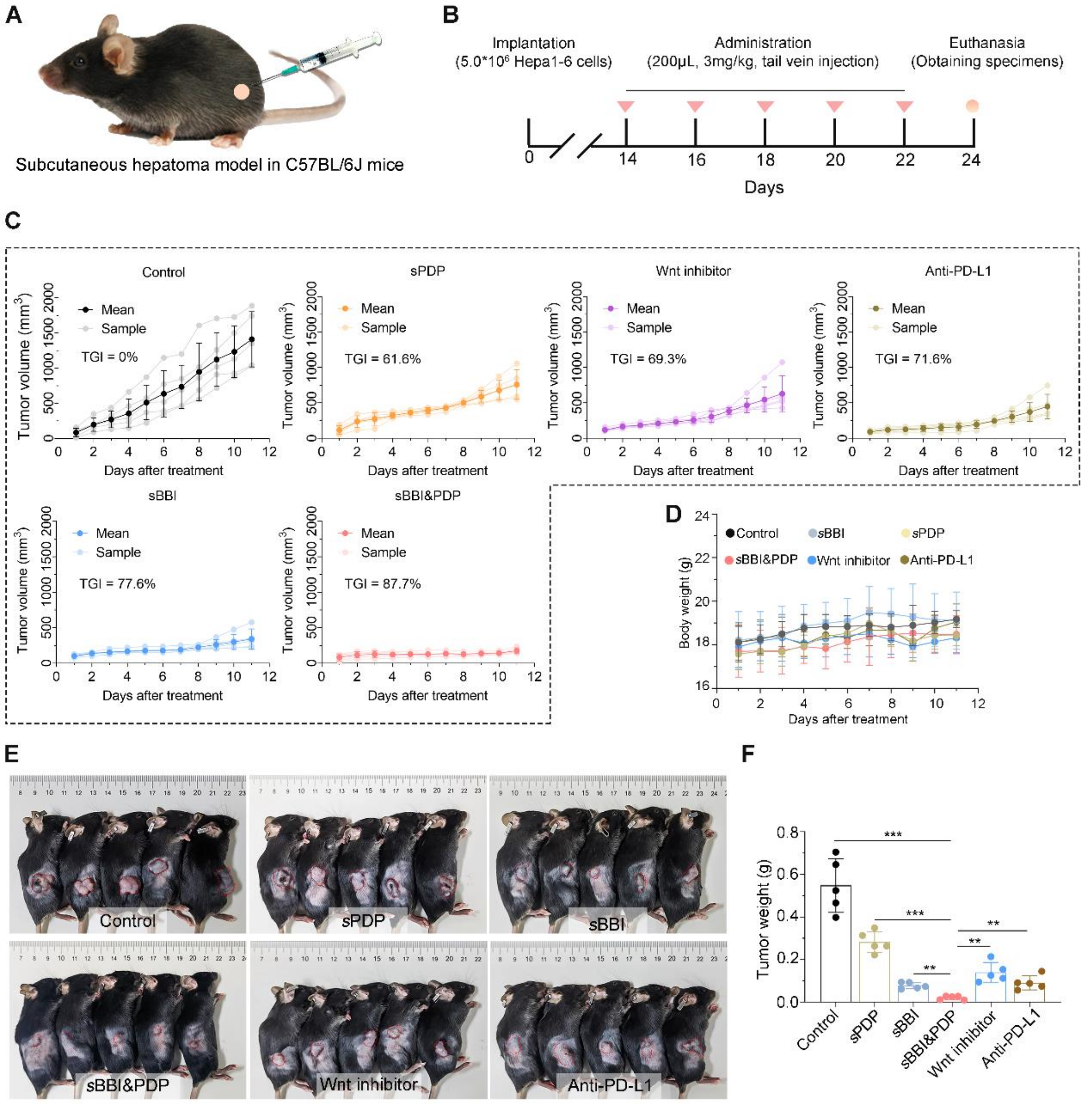

Figure S11. Anti-tumor efficacy of sBBI&PDP in subcutaneous HCC mouse model. (A) Schematic diagram of tumor model in C57BL/6J mice. (B) Dosage and schedule of administration (3mg/kg, 200μL). And the above mice were divided into 6 groups, respectively, Control, sPDP, sBBI, sBBI&PDP, Wnt inhibitor and Anti-PD-L1. (C) Growth curves of hepatoma with the indicated treatments (TGI, tumor growth inhibition; n = 5/group). (D) Body weight changes of each group of mice during the administration. (E) HCC images were shown and red circles indicated the subcutaneous tumor nodules. (F) Tumor weight of sBBI&PDP group were compared with Control, sPDP, sBBI, Wnt inhibitor or Anti-PD-L1 (n = 5, t-test, $^{**}p < 0.01$, $^{***}p < 0.001$).

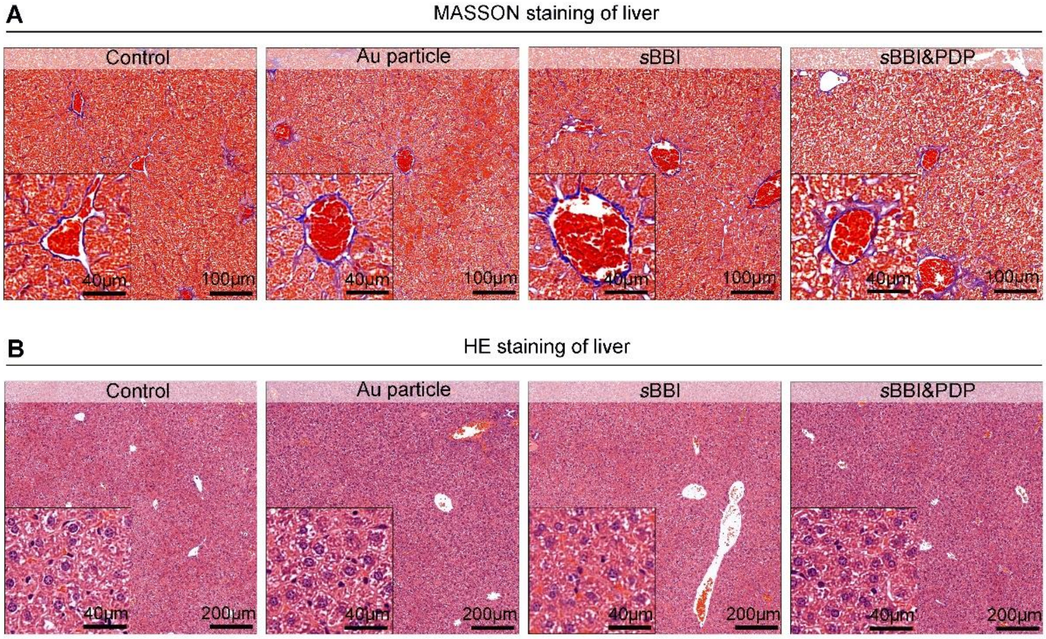

Figure S12. Safety of sBBI&PDP in liver. (A) MASSON and (B) H&E staining of representative liver parenchyma sections in C57 mice after exposure to 3mg/kg *s*BBI&PDP during the 12-days administration compared with Control, Au particle or *s*BBI.

2. Experimental section

General remarks

$HAuCl_4 \cdot XH_2O$ was purchased from Aladdin Bio-Chem Technology Co., Ltd (Shanghai), and all sources of synthetic peptides were purchased from CS Bio Co., Ltd (Shanghai). All other chemicals used in this study were purchased from Sigma-Aldrich unless otherwise stated. Acetonitrile and water (HPLC grade) were from Fisher Scientific Ltd. All products were used as received without further purification.

Synthesis of therapeutic peptides (BBI-SH and PDP-SH)

Sequence of BBI-SH: KFERQKILDSQEQLEHRERSLQT-(Hle)-RDIQRML-(2-Nal)-PC

Sequence of PDP-SH: CHEHEKFEQR-(PEG3)-nyskptdrqyhfrr

All peptides were synthesized on appropriate resins on an CS Bio 336X automated peptide synthesizer using the optimized HBTU activation/DIEA in situ neutralization protocol developed by an HBTU/HOBT protocol for Fmoc-chemistry SPPS. After cleavage and deprotection in a reagent cocktail containing 88% TFA, 5% phenol, 5% H2O and 2%TIPS, crude products were precipitated with cold ether and purified to homogeneity by preparative C18 reversed-phase HPLC. The molecular masses were ascertained by electrospray ionization mass spectrometry (ESI-MS).

Fabrication of sBBI&PDP

An aqueous solution of tetrachloroauric acid ($HAuCl_4 \cdot XH_2O$, 1 mL, 10 mM) was mixed with 9 mL 50 mM HEPES buffer (pH 7.4, 50 mM). After 10min magnetic stirring, the solution color changed from golden yellow to wine red, which was the Au core. Meanwhile, add 1mL tetrachloroauric acid (10 mM) solution into 9 mL peptide buffer (50 mM HEPES, 20% ethyl alcohol) dissolved 5mg peptides (BBI-SH and PDP-SH). After then, mixed the 10mL peptide-Au with the 10mL Au-core, following by a 10-min magnetic stirring. Finally, removed the excess reactants by dialysis tubing (cutoff, 10 KDa) and washed twice by distilled water.

Physicochemical properties of sBBI&PDP

The morphology was observed on transmission electron microscopy (TEM), which was performed on an HT7700 operated at an acceleration voltage of 100 kV. The hydrodynamic size distribution (1 mg/mL in PBS, 1 mL) was obtained from the dynamic light scattering (DLS) measurement (Malvern Zetasizer Nano ZS system). For Zeta potential measurement, the nanoparticles (1 mg/mL, 1 mL) were incubated with PBS at different pH at 37 ºC for 30 min, and measured by dynamic light

scattering (DLS). The surface chemical structure of modified nanocrystals was evaluated by Fourier transform infrared (FT-IR) spectroscopy (Nicolet 6700) and UV−vis absorption spectra (Shimadzu 3000 spectrophotometer). X-ray photoelectron spectroscopy (XPS) analysis was performed by ESCALAB Xi+ (Thermo Fisher).

In vitro experiment

Cell culture and viability analysis

Hepa1-6 cells and RAW264.7 cells were purchased from ATCC, and maintained in Dulbecco's modified eagle's medium (DMEM) with 10% fetal bovine serum (FBS) and 1% penicillin-streptomycin (P/S) solution. The above cells were maintained in incubator (37°C，5% CO_2). The in vitro cell viability was measured by using a cell counts kit-8 (CCK-8, Thermo Fisher) assay in Hepa1-6 cells. Cells were plated in 96-well plates at a density of 2000 cells per well (100 μL). After 48 hours, cells were treated with prescribed samples at the indicated concentrations and times, respectively. And add 10 μL of CCK-8 to each well. Place the plate in a dark incubator for 4 hours. Absorbance at 450 nm was measured by Microplate Readers (Bio-Rad).

Cell apoptosis

According to the procedure above, cells in the vigorous cell proliferation phase were selected for apoptosis experiments. After Heap1-6 cells were digested and centrifuged, resuspend the cells and add 1 mL of PBS, pipette 10 μL of the cell suspension, place on a cell counting plate, and count under a microscope. Apoptosis experiments were performed using 6-well plates. Plate 200,000 cells in each well plate and add 2 mL of cell culture medium. After 48 hours of treatment, cells were detached with 0.25% trypsin without EDTA. Trypsin was neutralized in old medium. Cell suspension were collected in 5 mL flow cytometry tube and centrifuged at 1000 rpm for 5 minutes at 4°C. Discard the old medium, add 1 mL of pre-cooled PBS solution, gently suspend the cells and centrifuge again. After one repeat, discard the PBS solution, add 100 μL 10×Binding Buffer and mix gently. Annexin-V and 7-ADD (BD, Biosciences) were added with 5μL each and mixed in vortexes. The cells were incubated at room temperature and protected from light for 15min. The detection was completed on fluorescence activated cell sorting (FACS, BD Biosciences) analysis within 1h. Note that the whole process should be handled gently to reduce accident cell death.

Cell cycle

Hepa1-6 cells were maintained in 6-well plates and processed according to the prescribed groups.

Collect cells by digestion and centrifugation, wash with PBS, add 1 mL of pre-cooled 70% ethanol, mix gently, fix at 4°C for 24 hours, and centrifuge at 2000rpm at 4°C for 5 minutes. Discard the iced ethanol, add 1 mL of pre-cooled PBS, resuspend and centrifuge at 1000 rpm for 5 minutes at 4 °C. Discard the iced ethanol, add 1 mL of pre-chilled PBS, resuspend the cells and centrifuge at 1000 rpm for 5 min at 4 °C. Pour off the old solution and add 100 μL of propidium iodide (BD, Biosciences) to each sample - add the staining solution and mix the suspended cells slowly. Incubate at 25°C in the dark for 15 minutes, and detect the cell cycle by FACS within 1 hour. Protect from light during this process.

Colony formation

The 500 Hepa1-6 cells were inoculated into each well of a 6-well cell culture plate in equal amounts, added 2ml of medium, and cultured in a cell culture incubator. After the cells adhered to the wall, the experimental group and the Control group were intervened. Continue to grow in the cell culture incubator for 7-10 days, replace the cell solution every 3 days and observe the cell state, pay attention to the central cell clone, until the maximum number of single clone cells exceeds 50 cells, discard the medium, wash 2 times with PBS, fix with 4% paraformaldehyde solution for 15 minutes, stain with crystal violet for 30 minutes, and rinse with tap water. Dry the 6-well cell cloning plate, take pictures with a digital camera, and count cloned cell clusters under a microscope.

Western blotting and detection

The culture and counting of Hepa1-6 cells were the same as before the experiment. The 200,000 cells were seeded into each well of a 6-well cell template. After the cells adhere to the cell wall, the appropriate dose of drug is added. After 48 hours, discard the old medium, place on ice, wash twice with PBS, add 100 μL pre-prepared cell protein lysate (RIPA:PMSF = 100:1) to each well, shake well and place on ice to lyse 30 minutes. Cell debris and proteins were then collected and centrifuged at 13,000 rpm, 4 °C for 10 minutes in 1.5 mL EP tubes. After centrifugation, carefully collect the supernatant and transfer to a new EP tube. Take 2 μL of protein for protein quantification. Add the remainder directly to 1/4 volume of 5* Loading Buffer, denature the protein at 100°C for 10 min, and store in a -20°C freezer. Quantification was performed by the Bicinchoninic Acid assay. Perform WB electrophoresis, transmembrane and capping according to the product specifications. Primary antibodies to GAPDH (Proteintech, 60004-1-Ig), PD-L1 (Proteintech, 66248-1-Ig) or β-catenin (Abcam, ab32572) were then incubated overnight in a 4°C refrigerator. Incubate with secondary

antibodies for 2 hours at room temperature. After the enhanced chemiluminescence working solution was added to the protein film, the gel imaging system was used to observe and collect images, and the gray value of the protein was calculated using Image Lab software (Bio. Rad).

Cellular uptake

Hepa1-6 cells are seeded in 6-well plates at an appropriate density, approximately 200,000 cells per well. After cell attachment, 1 mL of drug was labeled with 10 μL of FITC (100:1). Keep away from light, and co-incubate the drug with the cells for 10 minutes. Digest cells according to normal procedures. After centrifugation, wash 2-3 times with PBS and resuspend in 150-200 μL PBS for FACS detection. FITC was incubated with drugs for 30 minutes. Plate with 6 cm confocal laser dishes, 100,000 cells per well. After cell attachment, FITC-labeled drug was added. Drugs were co-cultured with cells for 6 h. Cells were washed twice with PBS and cellular uptake observed under a confocal laser fluorescence microscope.

In vivo experiment

All animal experimental procedures were performed in accordance with institutional guidelines and approved by the Laboratory Animal Center of Xi'an Jiaotong University.

The orthotopic homograft mice model of hepatocellular carcinoma

Orthotopic hepatocellular carcinoma (HCC) model were used in 5-week-old female C57BL/6J mice (Beijing Vital River, P.R. China). Prepare Hepa1-6 cells in the logarithmic growth phase in advance, use sterile PBS to make a single cell suspension, the concentration is 2×10^7/mL, 1 million cells per mouse, and the injection volume is 50 μL. Mice were anesthetized with 0.8% pentobarbital, 60 mg/kg, by intraperitoneal injection. After anesthesia, the mice were fixed on the operating board, and the surgical site was disinfected. A longitudinal incision was made about 1 cm below the xiphoid process of mice to open the abdominal cavity, and the left lobe of the liver was gently exposed with a sterile cotton swab. Fix the liver lobe with a cotton swab in the left hand, insert a 1mL micro-syringe along the liver surface at 15-30° with the right hand, penetrate into the liver about 0.5cm, inject the liver cancer cell suspension slowly, withdraw the needle slowly, and use a sterile cotton swab to lightly press the injection site to stop bleeding. The liver was then carefully placed back into the abdominal cavity, the abdomen was sutured layer by layer, and the incision was sterilized. Place the mice on an electric blanket until the mice wake up, then return them to their cages and observe the changes in the mice's vital signs and body weight. The orthotopic tumorigenesis time

of mouse HCC is about 2 weeks.

Anti-tumor efficacy of *s*BBI&PDP in an orthotopic homograft mice model of HCC

The model was constructed according to the mouse orthotopic liver cancer model, with 5 mice in each group, divided into 5 groups: Control, *s*BBI&PDP, *s*BBI, Au particle, Anti-PD-L1. Two weeks after tumor formation, the dose was 3 mg/kg, the volume was 200 μL, administered once every 2 days, 5 times in total. The body weight changes of the mice during the administration were recorded. At the end of the dosing period, tumor-bearing mice were euthanized. The mouse liver and tumor were taken out, photographed, the number of tumor nodules was recorded, and the weight of the liver tumor was weighed. Liver tumors were fixed with 4% paraformaldehyde, dehydrated and embedded in paraffin. Flakes with a thickness of 5 μm were then produced. Used for H&E staining or immunohistochemical staining to evaluate histopathological changes after drug treatment of liver cancer.

Immunohistochemical (IHC) staining

Sections were cut to 5 μm thickness, deparaffinized and rehydrated. Endogenous peroxidase activity was blocked with hydrogen peroxide/methanol and antigen was recovered by autoclaving in Tris-EDTA buffer solution (pH 9.0) for 10 minutes. The resulting tissue sections were then incubated overnight at 4°C with antibodies against β-catenin (Abcam, ab32572), Cyclin D1 (CST, E3P5S), PD-L1 (Proteintech, 66248-1-Ig) or Ki-67 (CST, 12202T), respectively. After 15 minutes incubation with labeled streptavidin-biotin complex, slides were stained and visualized using the Slide Viewer software (3DHISTECH, Hungary). Each stained section was scored by at least 5 randomly selected × 10 and × 50 high-resolution fields for further statistical analysis.

Double immunofluorescence staining of $CD3^+$/$CD8^+$ and $CD4^+$/$CD25^+$ T cells

The primary antibodies comprised a mixture of two antibodies: CD3 (Proteintech, 17617-1-AP) / CD8 (Abcam, ab22378) or CD4 (Abcam, ab183685) /CD25 (Abcam, ab128955). For the secondary antibody, a mixture of Alexa Fluor Cy3-conjugated goat anti-mouse IgG and Alexa Fluor 488-conjugated goat anti-rabbit IgG was used in this experiment. Double-stained HCC tissue shown in the immunofluorescence program, manually counted in 5 fields at original magnification of 400×.

Proteomics sequencing and analysis

Proteomic samples were obtained from mouse hepatoma tissues after 5 times administration (once every 2 days) with *s*BBI&PDP (3 mg/kg), Anti-PD-L1 (3 mg/kg) or normal saline (200 μL). Liver

tumor tissues were collected on ice, frozen in liquid nitrogen and stored in a −80 °C freezer. And then the tissues were thawed on ice at 4°C and lysis buffer (8 M Urea, Bio-Rad) was added to extract the protein at 1:10 weight : volume. The concentration of protein was quantified by Bradford protein assay (Thermo Fisher). According to the quantitative results, 100 μg of protein was extracted for enzymatic hydrolysis and desalting. Dried peptides were dissolved in 0.1% formic acid (FA), centrifuged at 14,000g for 10 minutes, and peptide concentrations were estimated from A_{280} nm absorbance using a Thermo Scientific Nanodrop One. Liquid chromatography-mass spectrometry (LC-MS) analysis was performed on a Q Exactive HF-X mass spectrometer (Thermo Fisher) coupled to a Nano-electrospray Flex ion source (Thermo Fisher) and an Easy-nLC 1200 (Thermo Fisher). The RAW data files were processed and quantified by Proteome Discoverer software v2.4.1.15 (Thermo Fisher), and searched against the [RefSeq Human protein database (24078 sequences, release 2017_03)] using the SEQUEST algorithm. For quantification, proteins with at least one unique peptide were considered for further analysis. Student *t*-test was used to identify proteins with significantly different expression. Volcano plots and Gene Set Enrichment Analysis (GSEA) were created using the Qlucore Omics Explorer 3.2. Pathway analysis was performed using Ingenuity Pathway Analysis software.

HCC patient-derived xenograft model in hu-PBMC-NSG mice

Xenograft tissue from a patient with advanced HCC was inoculated into the axilla of 5-week-old female NSG mice (Beijing Vital River, P.R. China) labeled with PDX-P_0. About 2 months later, when the tumor volume reached about 1000 mm^3 (Tumor volume =1/2×length×width^2), P_0 tumor tissue was collected for passage experiment in NSG mice, and PDX-P_1 labeling was performed. As above, the P_1 tumor tissue was obtained and named as PDX-P_2 for formal experiments. After about 4 weeks, when the tumor volume was 50-100 mm^3, 5 mice in each group were divided into 5 groups: Control, *s*BBI&PDP, *s*BBI, Au particles, Anti-PD-L1. Human peripheral blood mononuclear cells (PBMCs) were inoculated at a dose of 750,000 cells/200 μL and recorded as day 0. Control (NS), *s*BBI&PDP, sBBI, Au particle and Anti-PD-L1 were injected at a dose of 3 mg/kg and a volume of 200 μl on days 2 and 4, respectively. After 1 day of rest, PBMCs were re-injected and repeated for administration of 4 consecutive cycles. During the dosing period, body weight changes and tumor growth of the mice were recorded. TGI = [1-RTV (experimental group)/RTV (Control group)] ×100% (TGI, tumor growth inhibition value. RTV, relative tumor volume. RTV = V_t/V_0. V_t, tumor volume

at the end of the experiment. V_0, tumor volume at the beginning of the experiment). After euthanasia of NSG mice, liver tumors were collected for histopathology, and the staining procedure for tissue sections was as described above.

Toxicity studies

Thirty 8-week-old female C57BL/6J mice (Beijing Vital River, P.R. China) were selected for drug safety testing, six in each group, and divided into 5 groups (Control, *s*BBI&PDP, *s*BBI, Au particle and Anti-PD-L1). The Control group received 200 μL of normal saline (NS), and the other 4 groups received a dose of 3 mg/kg and a volume of 200 μL. Solution administration was proceeded by using a visual injection device of mouse tail vein. The test duration was 12 days with administration every 2 days. After the experiment, the mice were proceeded by euthanasia, and the whole blood of the mice was collected for blood routine examination, and the serum of the mice were performed for liver and kidney function tests. The tissue sections of mouse heart, liver, spleen, lung and kidney were stained with hematoxylin and eosin (H&E) to observe the toxicity of each organ.

Statistics

Statistical analysis was performed using Graphpad Prism 8.0 software. Comparison among groups were analyzed using Student's *t*-test or One-way analysis of variance. Survival curves (median survival time) were analyzed using the Kaplan-Meier method.
